# Identification of a functional docking site in the Rpn1 LRR domain for the UBA-UBL domain protein Ddi1

**DOI:** 10.1186/1741-7007-9-33

**Published:** 2011-05-31

**Authors:** Tara A Gomez, Natalie Kolawa, Marvin Gee, Michael J Sweredoski, Raymond J Deshaies

**Affiliations:** 1 Division of Biology, Beckman Institute, California Institute of Technology, Pasadena, CA 91125, USA; 2 Proteome Exploration Laboratory, Beckman Institute, California Institute of Technology, Pasadena, CA 91125, USA; 3 Howard Hughes Medical Institute, California Institute of Technology, Pasadena, CA 91125, USA

## Abstract

**Background:**

The proteasome is a multi-subunit protein machine that is the final destination for cellular proteins that have been marked for degradation via an ubiquitin (Ub) chain appendage. These ubiquitylated proteins either bind directly to the intrinsic proteasome ubiqutin chain receptors Rpn10, Rpn13, or Rpt5, or are shuttled to the proteasome by Rad23, Dsk2, or Ddi1. The latter proteins share an Ub association domain (UBA) for binding poly-Ub chains and an Ub-like-domain (UBL) for binding to the proteasome. It has been proposed that shuttling receptors dock on the proteasome via Rpn1, but the precise nature of the docking site remains poorly defined.

**Results:**

To shed light on the recruitment of shuttling receptors to the proteasome, we performed both site-directed mutagenesis and genetic screening to identify mutations in Rpn1 that disrupt its binding to UBA-UBL proteins. Here we demonstrate that delivery of Ub conjugates and docking of Ddi1 (and to a lesser extent Dsk2) to the proteasome are strongly impaired by an aspartic acid to alanine point mutation in the highly-conserved D517 residue of Rpn1. Moreover, degradation of the Ddi1-dependent proteasome substrate, Ufo1, is blocked in *rpn1-D517A *yeast cells. By contrast, Rad23 recruitment to the proteasome is not affected by *rpn1-D517A*.

**Conclusions:**

These studies provide insight into the mechanism by which the UBA-UBL protein Ddi1 is recruited to the proteasome to enable Ub-dependent degradation of its ligands. Our studies suggest that different UBA-UBL proteins are recruited to the proteasome by distinct mechanisms.

## Background

Protein degradation via the ubiquitin proteasome system (UPS) is one of the cell's tools for selective negative regulation of intracellular proteins. Degradation via the UPS has roles in maintaining protein quality control, signaling, and cell cycle progression [1,2]. Ubiquitin is a small protein that is highly conserved in eukaryotes and is the crux of the UPS system. The UPS system is built upon three classes of enzymes--E1, E2 and E3- that act sequentially to build ubiquitin chains on protein substrates. Once a protein substrate has been modified by a chain of at least four ubiquitins, it is then degraded by the 26S proteasome in an ATP-dependent manner [3,4].

The proteasome is a 33-subunit protein complex that is involved in turning over a minimum of 20% of the yeast proteome (SCUD; http://scud.kaist.ac.kr/index.html). Other lines of evidence suggest that the vast majority of cytoplasmic protein degradation is mediated by the proteasome [5]. The proteasome is composed of two main components: a 20S catalytic core particle (CP) and a 19S regulatory particle (RP). The 19S regulatory particle can be dissected into two sub-complexes, the lid and the base. The base sub-complex is composed of two non-ATPase subunits, Rpn1 and Rpn2, as well as six ATPase subunits (Rpt1 to 6) that are thought to unfold and feed substrate into the CP.

How ubiquitylated substrates converge onto the proteasome is an active area of research that has been studied with the greatest depth in *Saccharomyces cerevisiae*. So far, at least two independent mechanisms have been discovered. In the first case, the yeast proteasome contains two intrinsic receptors, Rpn10 and Rpn13, that contain defined ubiquitin binding domains [6,7]. Mammalian proteasomes contain a third intrinsic receptor, Rpt5 [8]. Rpn10 contains a highly conserved ubiquitin interaction motif (UIM), whereas Rpn13 binds ubiquitin via a pleckstrin motif that was not previously known to interact with ubiquitin [6,7]. Although neither Rpn10 nor Rpn13 is essential, *rpn10Δ *and *rpn13Δ *mutants exhibit phenotypes consistent with a role for these proteins in the docking of substrates to the proteasome. Rpt5 can be cross-linked to ubiquitin chains, but the means by which it binds ubiquitin and the genetic significance of this activity remain to be determined. Substrates may be able to bind the proteasome directly via these three intrinsic receptors.

In the second mode of delivery to the proteasome, receptors, including the budding yeast Rad23, Dsk2, and Ddi1 proteins, contain an N-terminal ubiquitin like domain (UBL) that binds to the proteasome and a C- terminal ubiquitin association domain (UBA) that binds to ubiquitin chains [9-12]. Unlike Rpn10 and Rpn13, these proteins are not stoichiometric subunits of the proteasome. Instead, it is thought that this class of proteins rapidly cycles on and off the proteasome [13], serving as 'shuttle' receptors that bind substrates in the cytoplasm and nucleus and deliver them to the proteasome. The UBA-UBL proteins dock at the proteasome by binding the largest subunit of the proteasome, Rpn1 [12,14,15], although recent evidence suggests that the UBA-UBL proteins also bind other subunits within the proteasome. For example, multiple lines of evidence suggest that in yeast Dsk2 may also be able to interact with Rpn10 and Rpn13, and yeast Rad23 may also bind Rpt6 [7,16-18]. Human Rad23 is also able to bind both human Rpn10 and Rpn13 [7] and in an NMR experiment, binding of yeast Rad23 to Rpn10 was observed [18].

While it is clear that substrates can use two different mechanisms to engage the proteasome, we still do not understand how substrates are allocated to one targeting pathway or the other. While there is evidence that some protein substrates utilize both the intrinsic and shuttling receptors [19], some proteasomal substrates are entirely dependent on either Rpn10 or Rad23 [20]. Moreover, although Rpn10 and Rpn13 are undoubtedly important receptors, electron microscopy and quantitative mass spectrometry data suggest that there are two populations of proteasomes, those containing and those not containing the intrinsic receptors [21-23]. Furthermore, deletion of *RPN10 *or *RPN13 *does not lead to profound deficits in cellular protein degradation [6,7,24]. Finally, while highly conserved [25], the UBA-UBL proteins are not essential for yeast cell growth [24,26,27]. Thus, although the proteasome itself is essential, none of the receptors that link substrates to the proteasome (with the exception of Rpt5) is essential. This has led to the assumption that targeting of substrates to the proteasome occurs by multiple, partially redundant mechanisms. Obtaining a clear understanding of how each pathway contributes to substrate recognition by the proteasome is of considerable importance given the central role of the UPS in regulatory biology and the clinical significance of the proteasome as a target for cancer therapy [28,29].

Rpn1, the largest subunit of the proteasome, contains nine repeat segments, known as leucine rich repeats (LRR), which adopt horseshoe-shaped structures that are thought to be generally important for protein-protein interactions [30]. The LRR domain of Rpn1 is thought to form a slightly open monomeric α-solenoid [31,32]. The first five contiguous repeat segments constitute LRR1, whereas the next four contiguous LRR repeats form LRR2. A 134 acidic amino acid stretch links LRR1 and LRR2 [15,33]. The minimal region sufficient for Rad23 binding to Rpn1 has been mapped to residues 417 to 628, which comprise LRR1 and an adjacent 21-residue acidic stretch on the C-terminal side. The UBL domains of Dsk2 and Ddi1 have also been shown to interact with the LRR domain of Rpn1 [14,15,34-36].

To gain a better understanding of how substrate delivery to the proteasome is controlled, we sought to identify an Rpn1 mutant that is defective in binding the UBA-UBL receptor proteins. We identified two mutations that disrupted binding of the UBA-UBL protein Ddi1 to the proteasome. Docking of Dsk2 to the proteasome was also moderately affected by these mutations in some genetic backgrounds. The delivery of ubiquitin conjugates to the proteasome was diminished in an *rpn1-D517A *single and even more so in an *rpn13Δ rpn1-D517A *double mutant. Lastly, we show that the *rpn1-D517A *mutation stabilizes the Ddi1 substrate, Ufo1.

## Results

### Rpn1^391 to 642 ^interacts with UBL domain proteins

**Figure 1:**
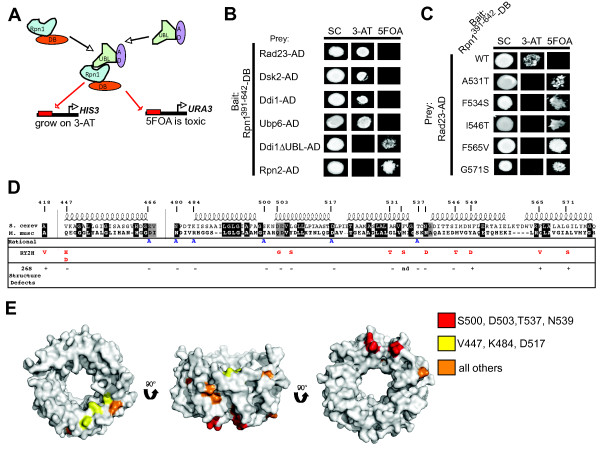
**Eighteen Rpn1 residues may be important for binding UBL domain proteins**. (**A**) The utilized yeast two hybrid system allows for both positive (growth on -HIS, -URA, +3-AT) and negative counter selection (growth on 5FOA) of UBL- Rpn1^391 to 642 ^interaction. **(B**) Rpn1^391-642 ^was sufficient for binding UBA-UBL proteins in a yeast two-hybrid system. Yeast cells were co-transformed with plasmids expressing Gal4-DBD fused to Rpn1^391 to 642 ^(Rpn1^391 to 642^DB) and Gal4-AD fused to either Rad23, Dsk2, Ddi1, Upb6, Ddi1ΔUBL or Rpn2. Protein-protein interaction is indicated by growth on 100 mM 3-AT and lack of growth on 0.2% 5FOA. (**C**) Representative *rpn1 *alleles found in the RY2H screen did not interact with Rad23 in the context of an Y2H experiment. (**D**) Sequence and secondary structure prediction alignments of yeast Rpn1 with mouse Rpn1 were made with MultiAlin http://multalin.toulouse.inra.fr/multalin/multalin.html using the model structure of Rpn1 [32]. Identical residues (black) and similar residues (gray) are indicated. Mutations identified in the RY2H that disrupt the interaction of Rpn1^391-642 ^with Rad23 and Dsk2 are indicated in red and rationally-designed mutations are indicated in blue. Mutant *rpn1 *alleles were plasmid shuffled into an *rpn1Δ *yeast strain and assayed for viability and proper 26S assembly. The positions of the identified mutations are indicated in the figure. A (-) indicates that assembly and viability were like wild type, a (+) indicates that we observed defects in proteasome stability (Figure S3) and (nd) indicates the strain was inviable. (**E**) The relative position of residues of interest from the RY2H screen and the rational sites chosen in (D) are shown on a model structure of Rpn1 proposed in reference 32. Residue A418 is not included in the model. The colors represent the residues indicated in the key.

To screen for mutations in Rpn1 that disrupt binding to UBA-UBL proteins, we engineered a reverse yeast two-hybrid system that reports on association between a fragment of Rpn1 (amino acids 391 to 642), a fragment including regions previously shown to be necessary and sufficient for UBA-UBL binding and four distinct UBL-containing proteins (Rad23, Dsk2, Ddi1, and the deubiquitinase Ubp6) known to interact with the proteasome [15,34,36-38]. Productive binding between Rpn1^391 to 642 ^and UBL proteins was expected to drive expression of *HIS3 *and *URA3*, resulting in growth on 3-aminotriazole (3-AT) and inability to grow on 5-fluoroorotic acid (5FOA) (Figure 1A). Growth assays revealed that Rpn1^391 to 642 ^was capable of binding to Rad23, Dsk2, Ddi1 and Ubp6 in yeast cells, whereas a Ddi1 fragment lacking its UBL domain and Rpn2, a proteasomal subunit, were unable to bind Rpn1^391 to 642 ^(Figure 1B).

### Identification of mutations in Rpn1^391 to 642 ^that block binding to UBL domain proteins

Using growth on 5FOA as a positive selection for loss of interaction between Rpn1^391 to 642 ^and UBL proteins, we screened a PCR-mutagenized allele library containing over 500,000 individual clones coding for Rpn1^391 to 642 ^and selected for mutants that could no longer interact with Rad23 (964 colonies were isolated) or Dsk2 (322 colonies). We screened these 1,286 transformants for their ability to reproduce their 5FOA^R ^phenotype. One hundred, ninety colonies that again tested 5FOA^R ^were sequenced. Forty-two of the sequenced 5FOA^R ^clones contained a mutation; single amino acid substitutions were identified in 32 clones, while silent mutations (4), and truncation or frameshift events (6) made up the remainder. Plasmids containing single Rpn1 mutations were then retransformed and assayed for their ability to reproduce the 5FOA^R ^phenotype. Twelve amino acid substitutions in 11 different residues of Rpn1 were identified as testing positive after being retransformed into our RY2H reporter strain (Figures 1C, D). Given that a structural model of the LRR region of Rpn1 exists [32], we also generated a panel of six 'rational' mutations that perturb residues predicted to be on the outside surface of the LRR domain (Figure 1D). The relative positions of the mutations discovered in the 'reverse yeast 2-hybrid' screen and the rational mutations are shown on the model structure of the Rpn1 LRR domain (Figure 1E).

### Mutant *rpn1 *alleles display synthetic growth defects in combination with ubiquitin receptor mutants

**Figure 2:**
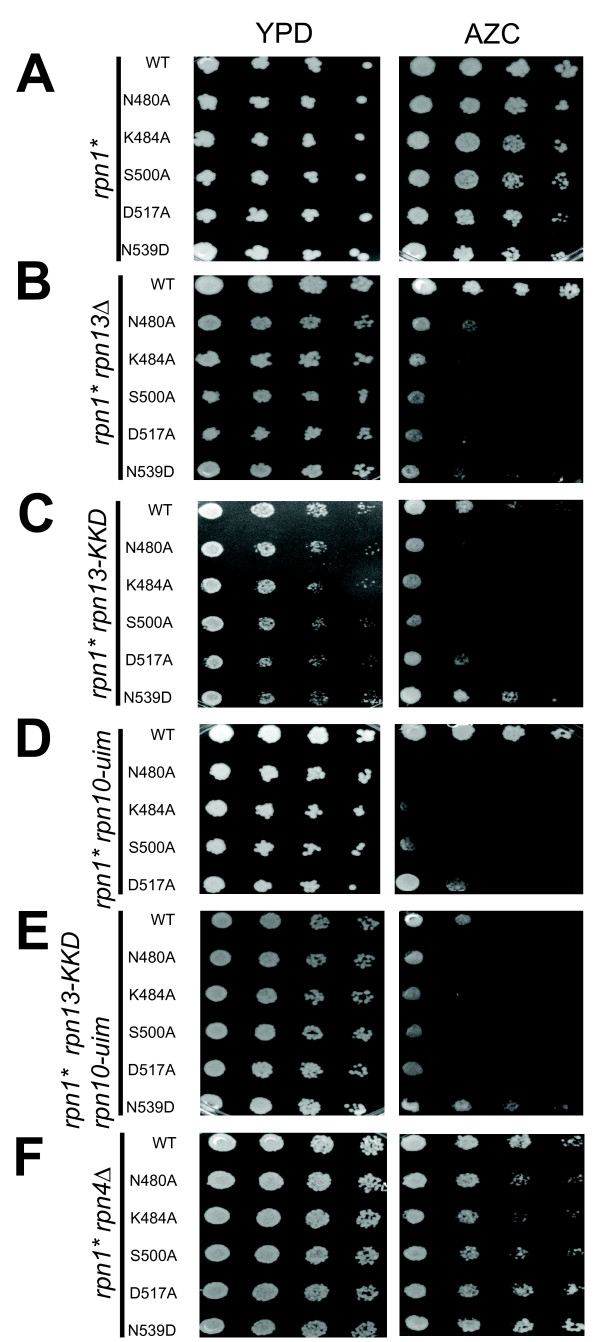
**Mutant rpn1 alleles display genetic interactions with mutations in genes for intrinsic ubiquitin receptors**. Five-fold serial dilutions of cells were plated onto the indicated media. The *rpn1 *mutants (rpn1*) were plasmid shuffled into an *rpn1Δ *strain containing (from top to bottom) either no additional mutations (A) or *rpn13Δ* (B), *rpn13-KKD* (C), *rpn10- uim* (D), *rpn13-KKD rpn10-uim* (E), or *rpn4Δ* (F). AZC refers to 5 mM of the proline analog l-azetidine-2- carboxylic acid (AZC)"

To evaluate whether any of the mutations in our panel of 18 substitutions had an effect on proteasome function, we reconstructed them into full-length *RPN1 *and performed a 'plasmid shuffle' to replace the essential *RPN1 *gene with each of our mutant alleles. A yeast *rpn1Δ *strain sustained by wild type *RPN1 *on a *URA3 *plasmid was individually transformed with a *LEU2 *plasmid bearing each mutant *rpn1 *allele and the cells were plated on 5FOA to identify clones from which the *URA3 *plasmid was evicted. We recovered 5FOA-resistant colonies from all transformants with the exception of *rpn1^F534S^*, indicating that 17 of our alleles retained at least partial *RPN1 *function. To evaluate the impact of our Rpn1 mutations on proteasome function, we plated cells on medium supplemented with the proline analog l-azetidine-2-carboxylic acid (AZC). Cells with defective proteasome function are sensitive to AZC [39,40], presumably because its incorporation into proteins causes misfolding, thereby placing an elevated demand on cellular quality-control pathways. As shown in Figure 2A and Additional file 1, Figure S1A, none of our mutants was hyper-sensitive to AZC.

Multiple receptors dock ubiquitinated substrates to the proteasome, including not only the UBL domain proteins but also Rpn10 and Rpn13 [7,20]. Unlike the other subunits of the proteasome, Rpn10 and Rpn13 are not essential. Therefore, we sought to test whether mutations in these receptors might sensitize cells to our *rpn1 *alleles. Deletion of *RPN13 *by itself did not cause sensitivity to AZC (compare top rows of Figures 2A, 2B). However, a subset of our *rpn1 *mutants (both rational and RY2H derived) exhibited striking sensitivity to AZC when combined with *rpn13Δ *(Figure 2B and Additional file 1, Figure S1B). To test whether this synthetic defect was due to the role of Rpn13 as an Ub receptor, the five *rpn1 *mutants showing the most striking phenotypes were introduced by plasmid shuffle into an *rpn13-KKD *strain that contains a triple point mutation that inactivates the ubiquitin binding domain [7]. Four of the five tested *rpn1 *alleles showed a similar synthetic growth defect in the *rpn13-KKD *mutant background (Figure 2C). These data indicate that our Rpn1 mutant proteins sensitized cells to loss of an intrinsic proteasome ubiquitin receptor.

Given the synthetic effects seen with *RPN13 *alleles, we sought to test whether the same subset of *rpn1 *mutants exhibited genetic interaction with *RPN10*. Rpn10 contains two domains: a VWA domain that appears to play a structural role and an ubiquitin-binding UIM domain. We used plasmid shuffle to introduce *rpn1 *alleles into a mutant, *rpn10-uim*, in which the UIM domain is inactivated by a cluster of point mutations [41,42]. Whereas neither the individual *rpn1 *mutants (Figure 2A) nor *rpn10-uim *(Figure 2D) was hypersensitive to AZC, the double mutants exhibited striking sensitivity (Figure 2D). Similarly, we also found that the same *rpn1 *alleles further sensitized an *rpn13-KKD rpn10-uim *strain to AZC (Figure 2E).

As a test for specificity, we introduced the same set of *rpn1 *mutations into an *rpn4Δ *background. Rpn4 is a transcription factor that promotes proteasome gene expression, and *rpn4Δ *mutants have reduced proteasome levels and show synthetic phenotypes with a number of mutations that impinge on proteasome function [43,44]. In contrast to the results seen with *rpn13Δ*, *rpn13-KKD*, and *rpn10-uim*, none of the five *rpn1 *mutants tested exhibited a synthetic AZC-sensitive phenotype when combined with *rpn4Δ *(Figure 2F). Taken together, these data suggest that the *rpn1 *mutant alleles impinge specifically on compromised receptor function, and do not cause general proteasome impairment.

### Recruitment of Ddi1, Dsk2 and ubiquitin conjugates to proteasomes is compromised in *rpn1-D517A and rpn1-K484A *mutants

**Figure 3:**
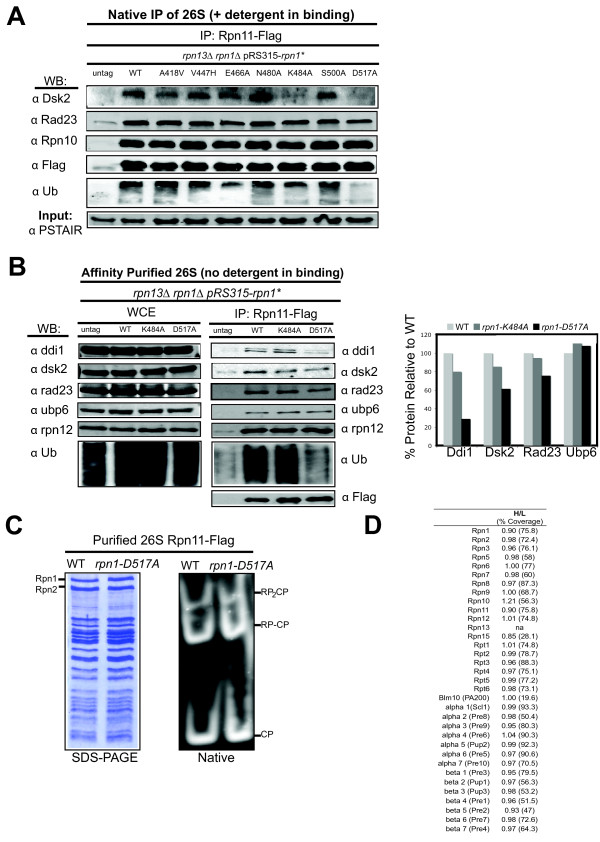
**Ddi1, Dsk2, and Ub conjugate recruitment to the proteasome is compromised in *rpn1-D517A *and *rpn1-K484A***. (**A**) Affinity-purified *rpn13Δ rpn1-K484A *and *rpn13Δ rpn1-D517A *proteasomes contain reduced levels of Dsk2. Detergent was present during the binding step of the anti-Flag immunoprecipiation as described in the Methods section. (**B**) Affinity-purified *rpn13Δ rpn1-D517A *proteasomes contain reduced levels of Ddi1 and Ub conjugates. Levels of UBA-UBL proteins, the lid subunit Rpn12 and polyubiquitin are shown for affinity purified proteasomes (IP) and in the whole cell extract input (WCE). This purification was performed in the absence of detergent. Densitometric quantification of the blot is shown (right panel). The amount of UBL protein was normalized to Rpn11^FLAG ^and wild type levels were set as 100%. (**C**) Proteasomes isolated from *rpn1-D517A *are intact. SDS-PAGE and native gel analysis of affinity purified 26S proteasomes from Rpn11-Flag tagged strains. The native gel was incubated with Suc-LLVY-AMC in the presence of ATP and 0.05% SDS to visualize RP and CP activity. The isoforms of the 26S proteasome are indicated. (**D**) Quantitative SILAC isotopic ratios are shown for all subunits of the proteasome isolated from an *rpn13Δ *strain (labeled with heavy isotopes; "H") in comparison to proteasomes isolated from an *rpn13Δ rpn1-D517A *strain (labeled with light isotopes; "L").

We next aimed to determine if any of the *rpn1 *mutations that showed genetic interactions with *rpn10-uim *and *rpn13-KKD *led to defects in recruitment of UBL containing proteins to the proteasome. To address this question, we first tagged *RPN11 *with sequences encoding the Flag epitope in a selection of *rpn13Δ rpn1 *mutants. We included *rpn13Δ *in this analysis due to potential redundancy between Rpn13 and Rpn1 for binding UBL domains. Proteasomes were immunoprecipitated from these strains and immunoblotted for the presence of UBL proteins. All double mutant proteasomes that were analyzed contained equivalent levels of associated Rad23, Dsk2 and Rpn10 except for *rpn1-D517A *and *rpn1-K484A*, both of which exhibited reduced levels of bound Dsk2 (Figure 3A). None of our *rpn1 *single mutants by themselves or in the *rpn10-uim *background showed significantly reduced levels of proteasome-bound Dsk2 (see for example the *rpn1-D517A *mutant in Additional file 2, Figure S2A, B; additional data not shown). To see if we could identify other binding-defective *rpn1 *mutations, we generated an additional set of 'rational' *rpn1 *alleles and tested them by using plasmid shuffle to introduce the alleles into an *RPN11^FLAG ^rpn1Δ rpn13Δ *background, followed by immunoprecipitation of the proteasomes and immunoblotting for UBL proteins. None of these mutants, which are listed in Additional file 3, Table S1, exhibited a greater UBL binding defect than the D517A or K484A alleles and so they were not pursued further.

Based on the proteasome association studies, we focused our attention on the K484A and D517A mutants. To evaluate the association of UBL proteins in greater depth, we retrieved proteasomes from both mutants (in an *rpn13Δ *background) and immunoblotted the immunoprecipitates to determine their content of Rad23, Dsk2, Ddi1, Ubp6, and total ubiquitin conjugates. The immunoblots are shown in the left panels of Figure 3B and densitometric quantification of the results is presented in the right panel. Proteasomes from both *rpn1 rpn13Δ *mutants contained normal or near-normal levels of the UBL proteins Ubp6 and Rad23. In this experiment the levels of Dsk2 were higher than those observed in Figure 3A, possibly because the immunoprecipitation was done under less stringent conditions. Interestingly, proteasomes recovered from *rpn13Δ rpn1-D517A *cells contained reduced levels of Ddi1 and total ubiquitin conjugates compared to proteasomes retrieved from either wild type or *rpn13Δ rpn1-K484A *cells. Similar results were obtained with proteasomes from *rpn1-D517A *and *rpn10-uim rpn1-D517A *cells (Additional file 2, Figures S2A, B). ). These results indicate that mutation of the D517 residue of Rpn1 by itself was sufficient to destabilize Ddi1 docking, and in combination with loss of Rpn13 modestly destabilized Dsk2 binding. We were intrigued by the mild defect in Dsk2 binding to *rpn13Δrpn1-D517A *proteasomes and questioned if combining *rpn10-uim*, *rpn13-KKD*, and *rpn1-D517A *mutations might yield a stronger defect in recruitment of Dsk2 since interaction of the UBL proteins has been observed with Rpn10 and Rpn13 [17,18]. We retrieved 10 proteasomes from both a double *rpn10-uim rpn13-KKD *and a triple *rpn10-uim rpn13- KKD rpn1-D517A *mutant and immunoblotted the immunoprecipitates to determine their content of Dsk2 and Ddi1. Proteasomes from an *rpn10-uim rpn13-KKD rpn1-D517A *strain contained fewer Ub conjugates in comparison to an isogenic strain containing wild type Rpn1 (Additional file 2, Figure S2C). Additionally, we quantified the change in the binding of Ddi1 and Dsk2 and again observed diminished recruitment of both proteins in the presence of the Rpn1-D517A mutation (Additional file 2, Figure S2C, right panel). This led us to investigate the effect of the Rpn1-D517A mutation in greater detail.

To determine whether Rpn1-D517A proteasomes were generally defective, we characterized them biochemically and found them to be completely normal by multiple methods. Purified Rpn1-D517A proteasomes exhibited a normal subunit composition when evaluated by SDS-PAGE (Figure 3C, left panel). Moreover, native nondenaturing gel electrophoresis verified that these proteasomes (Figure 3C, right panel) and those of all of the other strains indicated in Figure 1D (data not shown) were properly assembled and had normal chymotryptic activity. In fact, although we were manipulating the largest scaffolding subunit of the proteasome, only a small number of the mutations we studied had any negative consequences on proteasome stability (Figure 1D and Additional file 4, Figure S3). To characterize in detail the impact of the Rpn1-D517A mutation on proteasome composition, we performed a quantitative mass spectrometry technique, SILAC (stable isotope labeling with amino acid in cell culture). For this experiment,*rpn13Δ *cells were grown in medium supplemented with heavy isotopes of lysine and arginine while *rpn13Δ rpn1-D517A *cells were grown in medium with 'light' lysine and arginine. The two cultures were mixed immediately prior to lysis and proteasomes were purified by affinity chromatography on an anti-Flag resin. The purified sample was then analyzed by multidimensional mass spectrometry and the heavy/light ratios for peptides derived from proteasome subunits were determined (Figure 3D). This sensitive analysis confirmed that *rpn1-D517A *does not cause any apparent physical change in the proteasome.

While we did measure a slightly reduced level of Rad23 in *rpn13Δ rpn1-D517A *proteasomes by immunoblotting, our SILAC data indicated that the levels of Rad23 and Ubp6 were largely unaffected in *rpn13Δ rpn1-D517A *proteasomes, as they had heavy to light (H/L) ratios of 0.9 and 0.98 respectively. This is not unexpected, since it was reported in a prior SILAC study that the free and proteasome-bound pools of human Rad23 rapidly equilibrate in cell lysate [13]. Unfortunately, Ddi1 and Dsk2 peptides were not seen in our SILAC experiment. Capturing the association of all three UBA-UBL receptor proteins with proteasomes in native preparations is challenging, likely because these proteins interact very dynamically with the proteasome [13]. For instance, the association of Dsk2 with the proteasome has been reported to be difficult to capture [45]. Additionally, only one published mass spectrometry study has been able to simultaneously capture Rad23, Dsk2 and Ddi1 with the proteasome, and that study relied on chemical cross-linking to stabilize the association of dynamically-bound proteasome interactors [46].

### *In vitro *confirmation of a Ddi1 binding defect of Rpn13-deficient Rpn1-D517A mutant proteasomes

**Figure 4:**
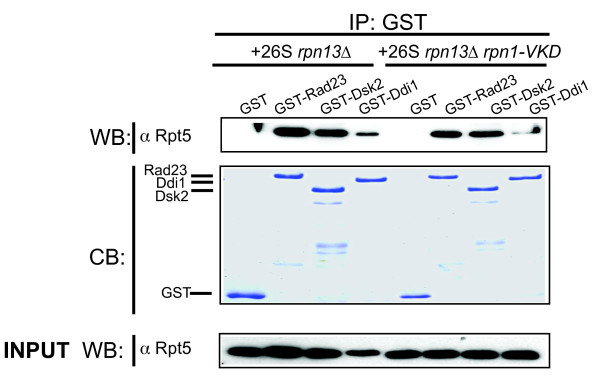
**Rpn1-D517A reduces binding of Ddi1 *in vitro***. GST-fused Rad23, Dsk2, Ddi1 and GST alone (as a negative control) were incubated with either proteasomes affinity purified from *rpn13Δ *or *rpn13Δ rpn1-V447H K484A D517A *(VKD) cells. The binding reactions were immobilized on glutathione resin, which was then washed and extracted with SDS-PAGE sample buffer. An Rpt5 immunoblot (upper panel) and a commassie stain to confirm equivalent recovery of the GST fusion proteins (middle panel) is shown. Inputs were immunoblotted with anti-Rpt5 and are also shown (lower panel). A qualitatively similar result was obtained in two independent experiments.

As an orthogonal approach, we sought to perform an *in vitro *binding assay that would confirm our analysis of proteasomes purified from mutant cells. Proteasomes were affinity purified from cells expressing Flag-tagged Rpn11 and incubated with recombinant GST-UBA-UBL proteins. Proteasomes affinity purified from *rpn13Δ *cells were successfully pulled-down by all three baits. However, Rpn13-deficient Rpn1-V447H K484A D517A (VKD) proteasomes exhibited strongly diminished binding capacity for Ddi1 (Figure 4). It should be noted that Rpn13-deficient Rpn1-V447H K484A D517A proteasome mutants behaved just as a Rpn13-deficient Rpn1-D517A mutant proteasomes in native immunoprecipitation experiments (Additional file 3, Table S1 and data not shown). However, it was surprising that we did not see a loss of Dsk2 binding to proteasomes isolated from an *rpn13Δrpn1-VKD *strain. It is possible that the effect of the D517A mutation on Dsk2 binding was subtle, and was overcome by mass action due to the high level of GST-Dsk2 employed in the pull-down.

### *rpn1-D517A *mutants exhibit a selective defect in protein degradation

**Figure 5:**
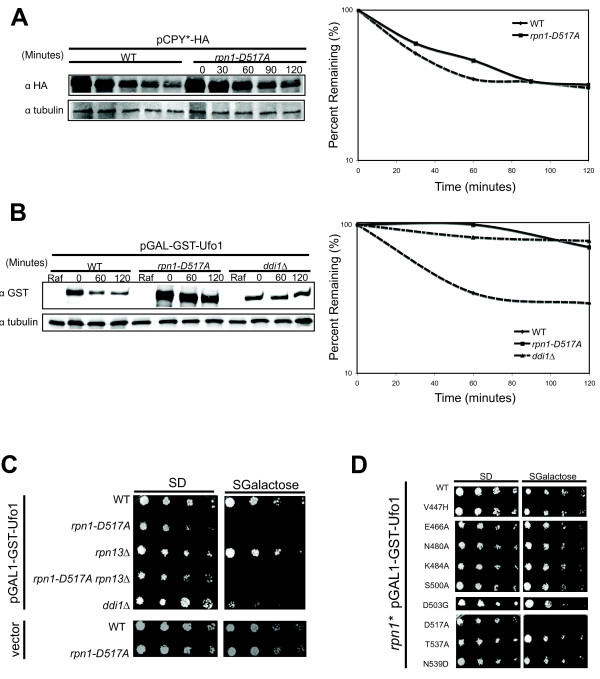
***rpn1-D517A *mutants exhibit a selective defect in protein degradation**. (**A**) Mutant *rpn1-D517A *cells degrade the Ufd1/Rad23/Dsk2 substrate CPY* with normal kinetics in a cycloheximide chase. Cycloheximide was added at time zero and samples were removed at the indicated time points for analysis by immunoblotting. Equal loading of extracts was confirmed by blotting with an anti-tubulin antibody (lower panel). The quantification of these blots is shown. (**B**) Ufo1 is stabilized in *rpn1-D517A *and *ddi1Δ *mutants. Wild type and mutant cells carrying a plasmid that expressed GST-Ufo1 from the GAL1 promoter were grown in raffinose medium and then induced with 2% galactose for 14 h. Dextrose was added at T_0 _to extinguish expression and samples were taken at the indicated time points for analysis by immunoblotting. Quantification is shown. (**C**) *rpn1-D517A *and *ddi1Δ *do not tolerate over expression of Ufo1. The indicated strains containing a plasmid that expressed GST-Ufo1 under the control of a galactose inducible promoter were grown on medium containing either glucose (SD, expression OFF) or galactose (SGalactose, expression ON). After two to three days, the plates were scored for growth. (**D**) Sensitivity of *rpn1-D517A *to GST-Ufo1 over-expression was specific and was not shown by other *rpn1 *alleles.

It is thought that UBA-UBL proteins exhibit some degree of selectivity in targeting specific substrates to the proteasome [20]. We hypothesized that the decrease of Ddi1 binding to proteasomes in an *rpn1-D517A *mutant might therefore result in turnover defects of substrates that are particularly reliant on Ddi1. In agreement with the normal binding of Rad23 and Dsk2 to the proteasome in an *rpn1-D517A *single mutant, no defect was seen in turnover of the Rad23/Dsk2-dependent substrate, CPY* (Figures 5A and Additional file 5, Figure S4B) [47], or the Dsk2 substrate Kre22 (Figure S4A) [48]. However, when we tested the Ddi1-dependent substrate, Ufo1 [49], we saw nearly complete stabilization in comparison to a wild type strain (Figures 5B and S4C). Note that the initial levels of plasmid encoded GST-Ufo1 were higher in *rpn1-D517A *compared to *ddi1Δ *cells. This was seen in two of three replicates. We do not know the basis for this. In agreement with the turnover data, we observed that over-expression of GST-Ufo1 was toxic to *ddi1Δ *and *rpn1-D517A *cells but not wild type cells (Figure C). This effect was exquisitely specific--neither *rpn13Δ *(Figure C) nor any other mutation in *rpn1 *that we tested (Figure 5D) conferred sensitivity to over-expression of DST-Ufo1.

## Discussion

Of the three UBA-UBL shuttle receptors linked to the proteasome, Ddi1 is the least studied and perhaps the most controversial. Prior data have established that Ddi1 binds polyubiquitin, albeit with lower affinity than Rad23 and Dsk2 [16,19,24,50]. However, while some studies report a physical interaction of Ddi1 with Rpn1 or the intact proteasome [14,24], there are a few reports that question the capacity for Ddi1 to bind the proteasome or Rpn1 [16,27,51]. The disparity in these reports may be due to the qualitative nature of immunoprecipitation experiments and the rapid dynamics of UBL binding to and dissociation from the proteasome [13]. Ddi1 has the most divergent UBL domain among the known UBA-UBL proteins, and hence, may have the weakest affinity interaction with the proteasome [27]. We have shown that Ddi1 is recovered with proteasomes immunoprecipitated from yeast cells, binds Rpn1 in a yeast two-hybrid assay, and binds to the proteasome in an *in vitro *pull-down assay. We have further validated these results by identifying an Rpn1 mutation that is selectively defective in binding Ddi1 and stabilizes the Ddi1-dependent proteasome substrate Ufo1. Hence, we conclude that Ddi1 does indeed interact with the proteasome in a specific and functionally-relevant manner. If Ddi1 binds the proteasome more weakly than other UBA-UBL proteins, which seems likely, it could explain why the D517A and K484A mutations reported here selectively disrupt interaction of Ddi1 with the proteasome.

Our study highlights the layered complexity of the interaction of shuttle proteins with the proteasome. With a single alanine substitution in the highly conserved D517 residue of Rpn1 we were able to significantly reduce the binding of Ddi1 to the proteasome. However, the interaction of other UBA-UBL proteins with the proteasome appears to be more complex. Recovery of Dsk2 with proteasomes was only mildly diminished in an *rpn1-D517A *mutant that also lacked *RPN13 *or the ubiquitin interaction motifs of both *RPN10 *and *RPN13*. Meanwhile, recovery of Rad23 was not affected appreciably by any mutation in Rpn1 analyzed during the course of this work. There are two possible explanations of these results. On the one hand, it is possible that the domains of these proteins have a gradient of affinity for Rpn1, with Ddi1 being the weakest binder and Rad23 the strongest. In this scenario, Rpn1-D517A may be a hypomorph that only modestly perturbs the UBL docking site, such that only the weakest binder (Ddi1) is excluded. We attempted to test this hypothesis by making numerous combinatorial mutations, (including a V447H K484A D517A triple mutant), none of which exhibited a substantially greater UBL binding defect than the D517A or K484A alleles (Additional file 3, Table S1). Thus, we do not favor the hypothesis that it is possible to disrupt recruitment of Rad23 and Dsk2 by mutating a single binding patch on Rpn1.

**Figure 6:**
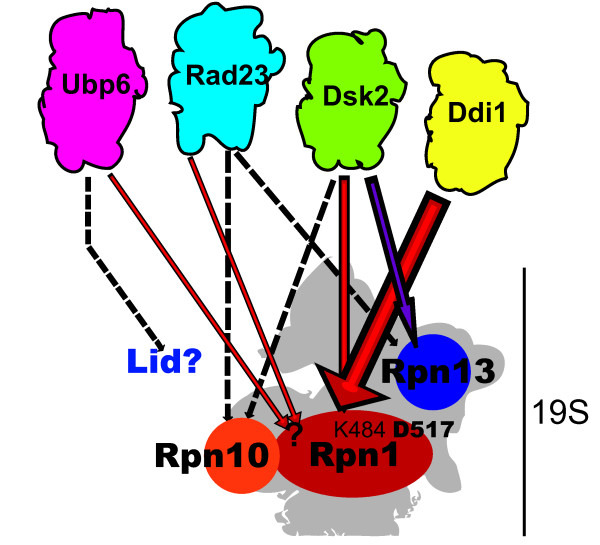
**Model for UBL protein interfacing with the proteasome**. Ddi1 shows a large dependence on the D517 residue of Rpn1 for binding to the proteasome. Additionally, deleting the intrinsic receptor Rpn13 Rpn13, or jointly the ubiquitin binding domains of Rpn13 and Rpn10, results in decreased binding of Dsk2 to the proteasome and reveals a role for the Rpn1-K484 residue in binding UBL proteins. However, Rad23 and the deubiquitinase Ubp6 did not show a dependence on residues D517 nor K484 of Rpn1. It is possible that Rad23 and Ubp6 interaction with the proteasome is stabilized by their interactions with other proteasomal subunits and/or other unidentified residues on Rpn1.

On the other hand, our model for UBL docking to the proteasome suggests that it is possible that Ddi1 uses only a single mechanism to bind the proteasome (direct binding to the LRR1 domain of Rpn1), whereas, in line with published reports [17,18], Rad23 and Dsk2 may use multiple mechanisms (including the binding site disrupted by the D517A mutation) and thus are more resistant to mutation (Figure 6). Our observation that reduction of Dsk2 binding was only seen in an *rpn1-D517A rpn13Δ *double mutant and more strikingly in a *rpn1-D517A rpn13Δ rpn10-uim *supports the idea that Dsk2 may be tethered to the proteasome by either Rpn1, Rpn13 or Rpn10 (Figure 6). The failure to see a significant reduction in binding of Rad23 in any single or double mutant may be due to there being multiple independent docking sites for Rad23 on the proteasome, although it should be noted that all of these docking sites appear to rely on the UBL domain [10,14,20,52]. Other studies have shown that Ubp6 may bind proteasome lid proteins while Rad23 may also bind Rpt6 [36,52], and that even Ub chains bound to Rad23 may contribute to its proteasome binding [53]. Biochemical data suggest that human Rad23 is recruited to the proteasome by the UIM domain of Rpn10 [54,55], and that even yeast Rad23 can bind Rpn10 [18] but it should be noted that this hypothesis has not been tested by genetic manipulation of Rpn10 in cells. Clearly, more work is needed to unravel the mechanisms underlying recruitment of the UBL domain proteins of Rad23, Dsk2, and Ubp6 to the proteasome.

## Conclusions

The current study identifies residues in the LRR1 domain of Rpn1 that contribute to shuttle receptor docking. We validate Ddi1 as a proteasomal shuttle receptor whose stable binding to the proteasome depends on Rpn1 residue D517. Consistent with this, D517 is also important for the degradation of a Ddi1-dependent substrate. We also show that in the absence of Rpn13, or the dual absence of the ubiquitin binding domains of Rpn13 and Rpn10, mutation of the D517 or K484 residues reduces the association of Dsk2 with the proteasome.

## Methods

### Yeast strains and growth conditions

Strains used in this study are listed in Additional file 3, Table S2. Listed strains are derivatives of the wild-type strain RJD 360 (W303 background). Standard yeast genetic techniques were used. Unless otherwise stated, strains were grown at 30°C and cultured on YPD.

### Plasmids

The *RPN1 *locus including 200 bp upstream and downstream of the ORF was amplified by polymerase chain reaction (PCR) from purified *Saccharomyces cerevisiae*genomic DNA using primers TG18 (GGGCGCCTCGAGGTTGACTATTTACAGCTCATC) and TG19 (GCGCCCGAGCTCAGCGCATCCATATTTACT). The resulting PCR product containing flanking *XhoI and SacI *restriction sites was digested with these enzymes and ligated into pRS315 and pRS316 CEN/ARS vectors. Silent mutations introduced by site-directed mutagenesis with oligonucleotides TG12 (gtcatttgtcaacgggttcttaaacctaggttattgtaacgataaattaat) and TG14 (gcagatgaagaagaaacggccgaaggacagacta) resulted in an *AvrII *restriction site at bp 1174 (amino acid 392) and an *EagI *site at bp 1920 (amino acid 640). Rpn1 mutations identified in the reverse two-hybrid screen or generated by the 'rational' approach were introduced into this construct by double digestion and ligation into the *AvrII *and *EagI *sites or by site mutagenesis. pEXP-Rpn1^391 to 640 ^was created by PCR amplification with primers TG1 (GGGGACA AGT TTG TAC AAA AAA GCA GGC TCTATGATGAACCTAGGTTATTGTAACGATAAA) and TG2 (GGG GAC CAC TTT GTA CAA GAA AGC TGG GTT TTCGGCCGTTTCTTCTTCATCTGCATC) and cloned using BP Gateway into pDONR-Express (Invitrogen, Carlsbad, CA, U.S.) and LR-cloned into pDEST-AD. *RAD23*, *DSK2*, *DDI1*, *UBP6*, and *RPN2 *were amplified by PCR and cloned into pDONR-Express and subsequently LR-cloned into pDEST-AD. All plasmids used in this study are listed in Additional file 3, Table S3

### *RPN1*^391-640 ^allele library construction

Rpn1 amino acids 391 to 640 were chosen as the target area to test for forward and reverse yeast two hybrid interactions. The pEXP-Rpn1^391 to 642 ^clone was used as a template for the allele library generation. Using attB primers TG4 (GGGGACA AGT TTG TAC AAA AAA GCA G) and TG5 (GGGGAC CAC TTT GTA CAA GAA AGCT), bp 1174 to 1926 were amplified by 25 cycles of PCR in 48 independent reactions, concentrated and gel purified. Approximately 150 ng of gel-purified product was BP-cloned into pDONR-Express and transformed via electroporation into TOP10 Electro-comp cells (Invitrogen). Plasmid DNA was collected from bacterial clones containing functional pENTR-Rpn1^391 to 642 ^clones. A yield of 500,000 clones was desired for good library coverage and this number was exceeded. Approximately 250 ng of purified pENTR-Rpn1 allele library DNA was LR-cloned into pDEST-DB and transformed via electroporation into *E. coli*. Again, over 500,000 colonies were pooled and the resulting pEXP-Rpn1 allele library DNA was purified.

### Forward and reverse yeast two-hybrid screen

The reverse yeast two hybrid assay was performed as described [56]. Briefly, the pEXP-DB-Rpn1 allele library was cotransformed with pEXP-AD-Rad23 or pEXP-AD-Dsk2 into the reporter strain MaV203 using the lithium acetate procedure. The transformation reactions were plated onto SC-Leu-Trp + 0.2% 5FOA. Plates were grown for approximately one week, and putative 5FOA^R ^colonies were picked and screened for reporter phenotypes. Interaction-defective alleles were tested for lack of activation of *GAL1-lacZ *and failure to grow on *SC-HIS*+3-amino-1,2,4-triazole (3-AT). Mild interaction-defective alleles showed some growth on 3-AT. pEXP-DB Rpn1 allele library plasmids were either purified or amplified by PCR from yeasts colonies that displayed 5FOA^R ^phenotypes and sequenced using primer 5'-GGC TTC AGT GGA GAC TGA TAT GCC TC-3'. Clones containing mutations were than retransformed into *MaV203 *and retested for proper reporter phenotypes. Direct PCR amplification of their pEXP-DB-Rpn1 insert was done as described [56]. Forward interactions were tested by assaying for growth on 50 mM or 100 mM 3AT and 0.1% or 0.2% 5FOA. The plates were scored between 24 and 72 hours.

### Plasmid shuffling of *rpn1 *alleles

*RPN1 *was replaced by *Kanmx6 *[57] by amplifying a cassette from pFA6a-KanMX6 using oligonucleotides TG20 (GGTCTACATAAGGTGCGATTCGTATAAATTTGGAAGACAATTGCAAGAAACGGATCCCCGGGTTAATTAA) and TG21 (GGTTTTGAATTTTTCCTATTCTGGTTGATATTGCCCAAAAGCTATTCAGTGAATTCGAGCTCGTTTAAAC). The PCR product was transformed into a diploid W303 strain (RJD381) creating strain RJD4166. This diploid strain was transformed with pRS316-RPN1 (RDB 2090), sporulated, and haploid segregants were selected for growth on G418 and SD-Ura. The resultant strain, RJD 4189 was used for plasmid shuffling. Plasmids were transformed into RJD 4189 and then transformants were selected for growth on 5FOA-containing media.

### 26S proteasome native gel analysis

Native gels were prepared and run as described [58]. Briefly, 2 mL of 5× native buffer (450 mM Tris base, 450 mM boric acid, 25 mM MgCl_2_, 2.5 mM EDTA (pH 8)), 0.9 ml 40% acrylamide/Bis solution (37.5:1), 7 ml H_2_O, 10 μl 0.5 M ATP, 90 μl 10% APS, and 9 μl TEMED were combined and allowed to set using the BioRad Mini-Protean Tetra gel system (Bio-Rad, Hercules, CA, U.S.)About 90 to 300 μg of protein supplemented with xylene cyanol and glycerol, was loaded per lane. Either purified proteasomes or cell extracts were run on native gels. Extracts were prepared as described [59]. Gels were run at 100V for 3.5 to 4 hours with 1× native buffer supplemented with 1 mM ATP. The gels were then soaked in 25 mL of developing buffer (50 mM Tris pH 7.5, 5 mM MgCl_2_, 1 mM ATP) followed by a 15 minute incubation at 30°C in substrate solution (50 mM Tris pH 7.5, 5 mM MgCl_2_, 1 mM ATP, 20 μM SVC LLVY AMC, 0.02% SDS). Cleavage of the fluorogenic substrate was visualized by exposure to UV light using an alphaimager.

### Native immunoprecipitaion of proteasomes for probing associated UBA-UBL proteins

Native immunoprecipitations were carried out as described [60]. Briefly, yeast cultures were grown to an OD_600 _between 1 and 2 in YPD and harvested by centrifugation. Pellets were washed in ice cold water and then flash-frozen in liquid nitrogen. Thawed pellets were resuspended in 1 mL of Lysis Buffer (composition described below) per 100 O.D. units. One milliliter of this lysate was mixed with an equivalent volume of glass beads and cells were disrupted by vortexing using the FastPrep-24 at a setting of 6.5 for 60 s, cooling on ice, and then repeating. Lysates were clarified by centrifugation at 14,000 rpm at 4°C for 15 minutes. Clarified supernatants were bound to anti-epitope beads for 1.5 hours at 4°C. The beads were washed four times with lysis buffer containing detergent (50 mM Tris, pH 7.5, 150 mM NaCl, 15% glycerol, 0.2% Triton X-100, 25 mM b-glycerophosphate, 25 mM NEM, 1× Protase Inhibitor tablet (minus EDTA), 0.5 mM AEBSF, 2 mM ATP, 5 mM MgCl_2_), and two times with buffer B (25 mM Tris pH 7.5, 10 mM MgCl_2_, 2 mM ATP). An equal bead volume of 2× SDS buffer was added prior to boiling for three minutes. Samples were resolved on 10% or 12.5% SDS-PAGE gels, transferred to nitrocellulose and immunoblotted. Antibodies used in this study are listed in Additional file 3, Table S4

### Purification of 26S proteasomes for immunoblotting

A total of 26S proteasomes were purified as described [61]. Briefly, Pre1-Flag (20S subunit) or Rpn11-Flag tag containing strains were grown as large-scale cultures (2 L), and lysed by grinding with a mortar in pestle in the presence of liquid nitrogen. Lysates were thawed in buffer A (50 mM Tris pH 7.5, 150 mM NaCl, 10% glycerol, 5 mM MgCl_2_, 5 mM ATP), bound to anti-Flag resin (Sigma, St. Louis, MO, U.S.), washed three times with buffer A supplemented with 0.2% Triton X-100, then washed two times with buffer B (25 mM Tris pH 7.5, 10 mM MgCl_2_, 2 mM ATP) prior to elution with Flag Peptide (Sigma).

### Turnover of CPY*HA and GST-Ufo1

For CPY*HA turnover, pCPY*HA/*URA3 *containing yeast strains were grown to an OD_600 _approximately 0.5, shifted to 37°C for one hour and then treated with 100 μg/ml cycloheximide, at which point a chase was initiated. Turnover of galactose-inducible Ufo1 was carried out as described [49]. Briefly, cells containing pEGH-Ufo1 (Open Biosystems, Huntsville, AL, U.S.)were grown overnight in SRaffinose-URA medium and diluted the next day to an OD_600 _0.2. At an OD_600 _approximately 1.2% galactose was added. Induction was for 14 hours. Cells were filtered and washed in YP and then resuspended in YP containing 2% dextrose. Samples were taken at intervals post dextrose addition, centrifuged, and flash frozen. Protein was extracted using boiling SDS-PAGE sample buffer, resolved by SDS-PAGE, transferred to nitrocellulose and immunoblotted. Blots were quantified by LI-COR Odyssey with IR dye-linked secondary antibodies (Invitrogen).

### Growth assays

For plating assays strains were grown overnight in YPD or SRaffinose-URA and diluted to an OD_600 _of 0.3 in water. Serial five-fold dilutions were prepared in water and spotted onto either YPD or minimal plates supplemented with various additives as described in the text. Plates were incubated at 30°C for two to three days.

### SILAC analysis of purified proteasomes

*RPN11^FLAG ^*yeast strains auxotrophic for lysine and arginine, were grown in either CSM with 2% dextrose containing 20 mg/L lysine and arginine or in "heavy" medium with 20 mg/L ^13^C_6_^15^N_2_-lysine and ^13^C_6_-arginine. Cells were grown to an OD_600 _of 2, harvested, and flash frozen before grinding in liquid nitrogen. Equivalent amounts of heavy and light cells were mixed 1:1 before proceeding with a proteasome affinity purification. Proteasomes were eluted in 8 M urea. After purification, Lys-C (Wako Chemicals, Richmond, VA, U.S.) was added for a four-hour digestion, followed by an overnight tryptic digestion in 2 M urea. The tryptic peptides were desalted on a C18 macrotrap (Michrom Bioresources, Auburn, CA, U.S.) and concentrated in a speedvac. Dried samples were resuspended and subject to StageTip based strong anionic exchange (SAX) as previously described [62]. Samples were eluted, concentrated, and then acidified with 0.2% formic acid prior to mass spectrometric analysis. All mass spectrometry experiments were performed on an EASY-nLC (Thermo Scientific, Waltham, MA, U.S.) connected to a hybrid LTQ-Orbitrap Classic (Thermo Scientific) with a nanoelectrospray ion source (Thermo Scientific). Peptides were resolved using a 240-minute gradient from 4% to 25% acetonitrile in 0.2% formic acid at a flow rate of 350 nl per minute. The mass spectrometer was operated in data-dependent mode to automatically switch between full-scan MS and tandem MS acquisition. All settings were as previously described [63]. Raw data files were analyzed by MaxQuant (v 1.0.13.13)

(MaxQuant, Matthias Mann Lab, Max Planck Institute, Germany; http://www.maxquant.org/) [64] and searched against the Saccharomyces Genome Database. The search parameters included tryptic digestion, a maximum of two missed cleavages, fixed carboxyamidomethyl modifications of cysteine, variable oxidation modifications of methionine, variable protein N-terminus acetylations, and a variable Gly-Gly tag on lysine residues with a 1% FDR thresholds for both peptides and proteins. At least two peptides were required for protein identification and at least two different scanning events were required for protein quantification.

### *In vitro *UBA-UBL proteasome binding assays

GST proteins were purified using standard methods and dialyzed into 50 mM Tris pH 7.5, 50 mM NaCl, 1 mM DTT, 10% glycerol. For co-immunoprecipitation experiments with UBA-UBL proteins and purified 26S proteasomes, 1 μM of GST or GST-fusion protein was mixed with 0.2 nM of 26S proteasome in the presence of IP buffer (50 mM Tris-HCl at pH 7.5, 150 mM NaCl, 1 mM EDTA, 1 mM DTT, 0.2% triton X-100, 10% glycerol, 10 mM MgCl_2_, and 5 mM ATP). The reaction was incubated with rotation for one hour at 4°C, after which point 30 μl of glutathione-sepharose beads were added to each reaction and reactions were incubated for another hour at 4°C. Beads were washed with 1 mL of IP buffer three times. Each sample was boiled in 2× SDS and loaded onto a 10% tris-glycine gel. Gels were both commassie stained and immunoblotted.

## Abbreviations

3-AT: 3-Amino-1,2,4-triazole; 5FOA: 5-fluoroorotic acid; AZC: Azetidine-2-carboxylic Acid; RY2H: reverse yeast two hybrid; Ub: ubiquitin; UBA: ubiquitin association domain; UBL: ubiquitin-like domain; UIM: ubiquitin interaction domain

## Authors' contributions

RJD and TAG designed and interpreted all of the experiments and wrote the paper. TAG also carried out all of the experiments. NK performed the mass spectrometry. MJS analyzed the mass spectrometry data. MG designed and performed the first repetition of the experiment in Figure 4. All authors read and approved the final manuscript.

## Supplementary Material

Additional file 1 **Figure S1**. Mutant *rpn1 *alleles derived from both the RY2H screen and rational mutagenesis display genetic interactions with mutations in genes that encode ubiquitin receptors intrinsic to the proteasome. Five-fold serial dilutions of cells were plated onto the indicated media. The *rpn1 *mutants (rpn1*) were plasmid shuffled into an *rpn1Δ *strain containing either no additional mutations (A) or *rpn13Δ (B). *AZC refers to 5 mM of the proline analog l-azetidine-2-carboxylic acid (AZC). In panel B, mutations derived from the RY2H screen are indicated with a red box. Click here for file

Additional file 2 **Figure S2**. Analysis of Ddi1 and Dsk2 association with proteasomes isolated from *rpn1-D517A *, *rpn10-uim rpn1-D571A, and rpn10-uim rpn13-KKD rpn1-D571A *mutants. (A) Affinity-purified *rpn1-D517A *proteasomes contain reduced levels of Ddi1 and Ub conjugates. Levels of UBA-UBL proteins, the lid subunit Rpn12 and polyubiquitin are shown for affinity purified proteasomes (IP) and in the whole cell extract input (WCE). (B) Affinity-purified *rpn10-uim rpn1-D517A *proteasomes similarly show diminished association of Ddi1 and Ub conjugates compared to *rpn10-uim *proteasomes. (C) Affinity-purified *rpn10-uim rpn13-KKD rpn1-D517A *proteasomes contain reduced levels of Ddi1, Dsk2 and Ub conjugates in comparison to proteasomes from an *rpn10-uim rpn13-KKD *strain. Densitometric quantification of this blot is shown on the right. The amounts of UBL proteins were normalized to Rpn11FLAG and wild type levels were set as 100%. Click here for file

Additional file 3 **Table S1**. Additional rational Rpn1 mutants used in this study. Supplemental Table S2. *S. cerevisiae *strains used in this study. Supplemental Table S3. Plasmids used in this study. Supplemental Table S4. Antibodies used in this study Click here for file

Additional file 4 **Figure S3**. Mutations at Rpn1 residues A418, N549, F565 and G571 render unstable proteasomes Pre1-myc13 tagged proteasomes from strains carrying plasmid borne Rpn1 alleles in an *RPN1 *null strain, were immunoprecipitated from whole cell extracts and analyzed by immunoblotting with the indicated antibodies. As shown, proteasomes with mutations at residues A418, N549, F565 and G571 exhibit dissociation of the 19S cap with the proteasomal base during immunoprecipitation experiments. Click here for file

Additional file 5 **Figure S4**. *rpn1-D517A *mutants exhibit a selective defect in protein degradation. (**A**) Mutant *rpn1-D517A *cells degrade the Dsk2 substrate Kre22 with normal kinetics. Strains carrying a plasmid that expressed GST-Kre22 from the *GAL1 *promoter were grown in raffinose medium and then induced with 2% galactose for three hours. Dextrose was added at time zero to extinguish expression and samples were taken at the indicated time points for immunoblot analysis. Below, cells were plated in five-fold serial dilutions onto either glucosoe or galactose containing medium and monitored for growth after two to three days at 30°C. (**B**) Mutant *rpn1-D517A *cells degrade the Ufd1/Rad23/Dsk2 substrate CPY* with normal kinetics in a cycloheximide chase. Cycloheximide was added at time zero and samples were taken at the indicated time points for immunoblot analysis. Equal loading of extracts was confirmed by blotting with an anti-tubulin antibody (lower panel). The quantification of these blots is shown in the right panel. This is a replicate of the experiment shown in Figure 5A. (**C**) Ufo1 is stabilized in *rpn1-D517A *and *ddi1Δ *mutants. Wild type and mutant cells carrying a plasmid that expressed GST-Ufo1 from the *GAL1 *promoter were grown in raffinose medium and then induced with 2% galactose for 14 h. Dextrose was added at T0 to extinguish expression and samples were taken at the indicated time points and analyzed by immuoblot. Quantification is shown in the right-hand panel. This is a replicate of the experiment shown in Figure 5B Click here for file
