# Supplementary figures and images for: Identification of a functional docking site in the Rpn1 LRR domain for the UBA-UBL domain protein Ddi1
Source: BMC Biol. 2011 May 31;9:33. doi: 10.1186/1741-7007-9-33 (PMC3126750; doi:10.1186/1741-7007-9-33)

Figure S1

**A**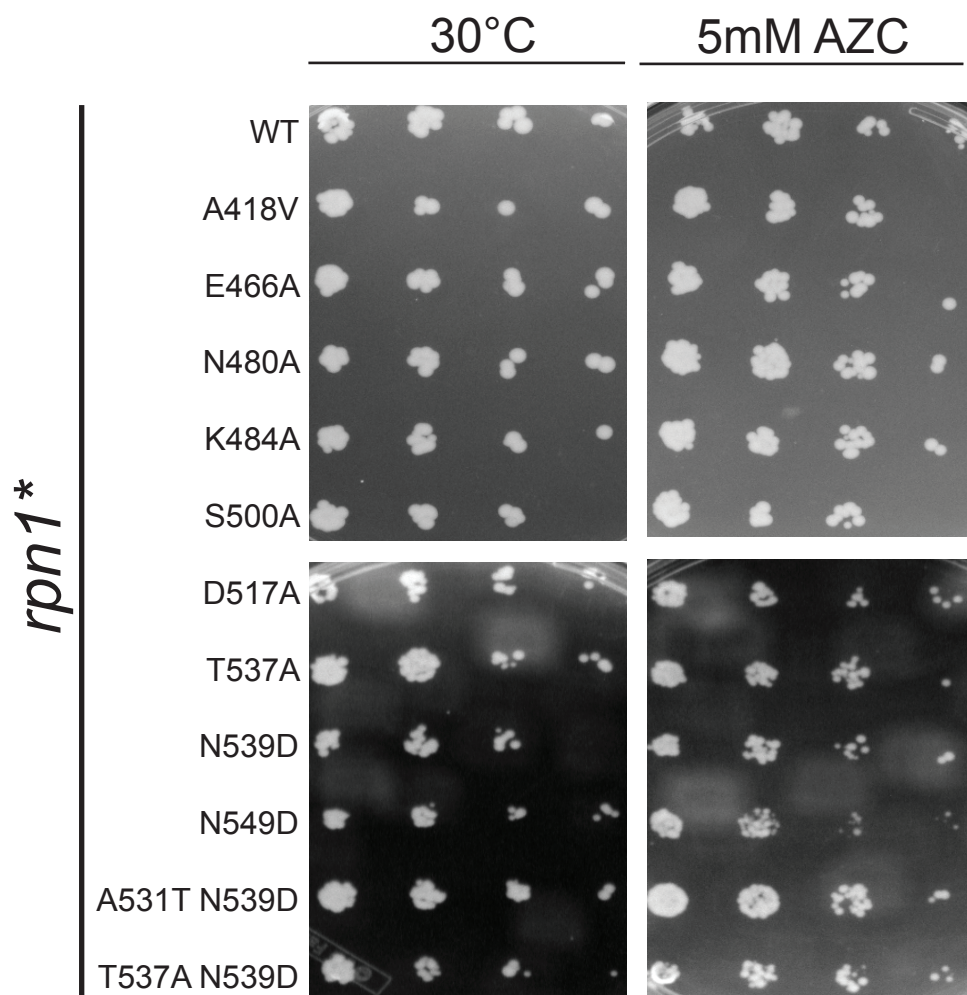**B**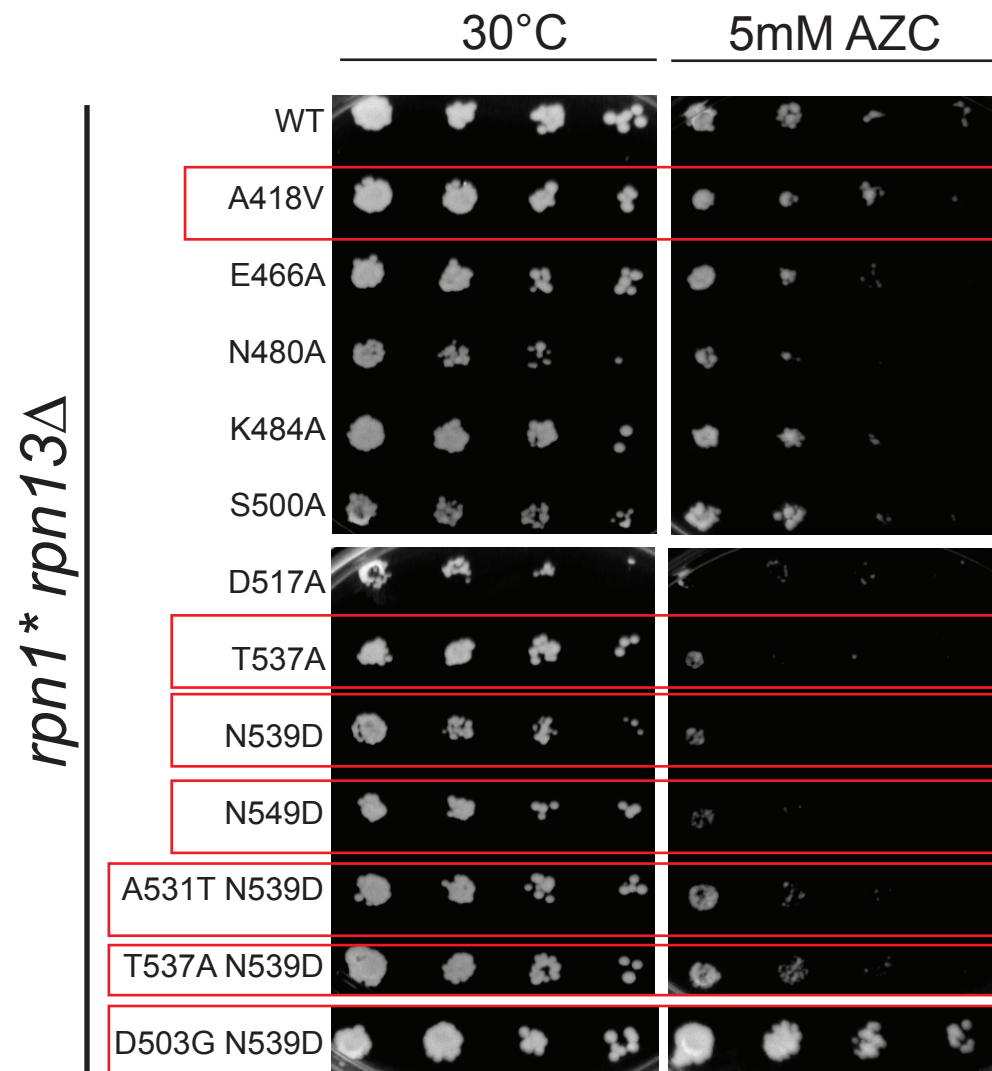

Supplement: Additional file 1 — Figure S1. Mutant rpn1 alleles derived from both the RY2H screen and rational mutagenesis display genetic interactions with mutations in genes that encode ubiquitin receptors intrinsic to the proteasome. Five-fold serial dilutions of cells were plated onto the indicated media. The rpn1 mutants (rpn1*) were plasmid shuffled into an rpn1Δ strain containing either no additional mutations (A) or rpn13Δ (B). AZC refers to 5 mM of the proline analog l-azetidine-2-carboxylic acid (AZC). In panel B, mutations derived from the RY2H screen are indicated with a red box. [file 1741-7007-9-33-S1.PDF]

Figure S2

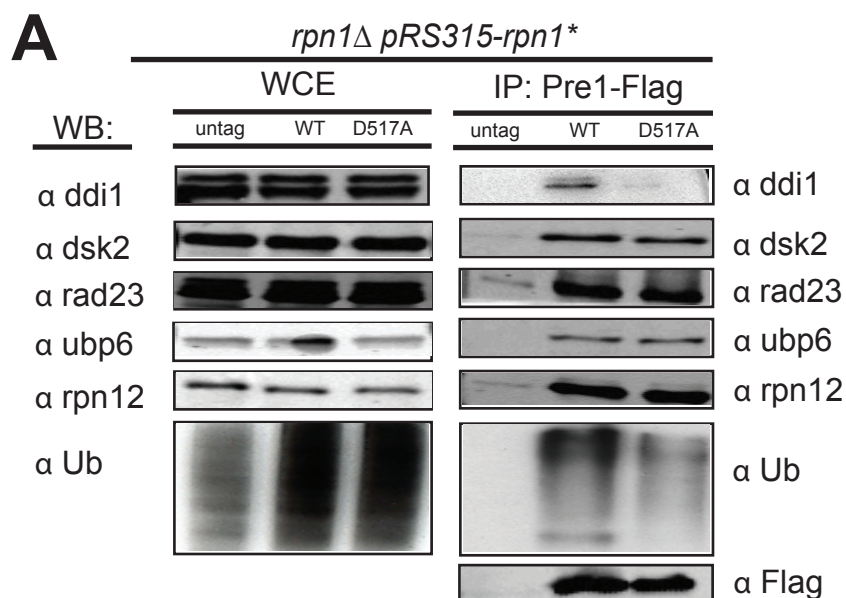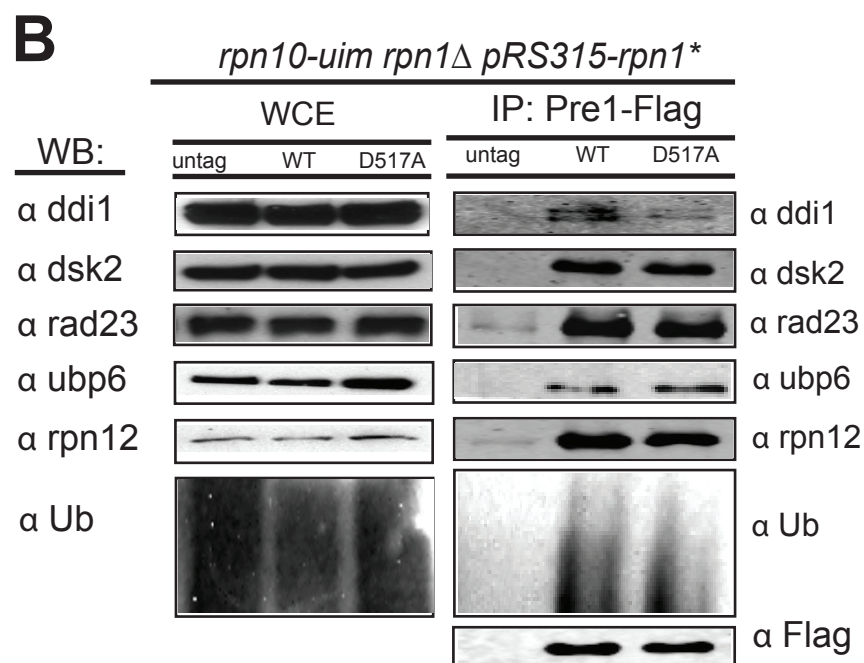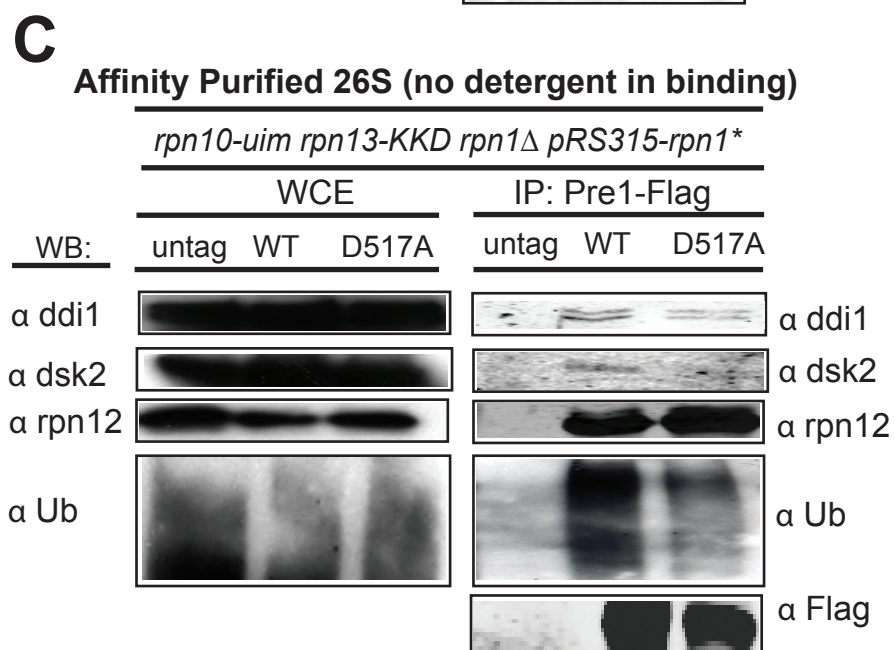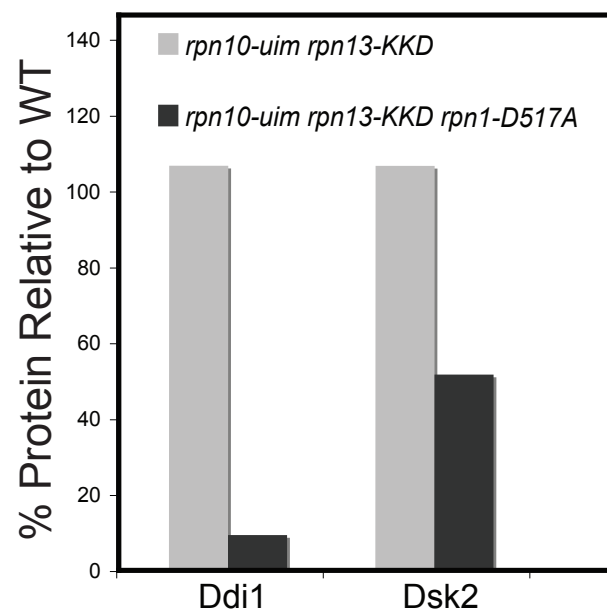

Supplement: Additional file 2 — Figure S2. Analysis of Ddi1 and Dsk2 association with proteasomes isolated from rpn1-D517A , rpn10-uim rpn1-D571A, and rpn10-uim rpn13-KKD rpn1-D571A mutants. (A) Affinity-purified rpn1-D517A proteasomes contain reduced levels of Ddi1 and Ub conjugates. Levels of UBA-UBL proteins, the lid subunit Rpn12 and polyubiquitin are shown for affinity purified proteasomes (IP) and in the whole cell extract input (WCE). (B) Affinity-purified rpn10-uim rpn1-D517A proteasomes similarly show diminished association of Ddi1 and Ub conjugates compared to rpn10-uim proteasomes. (C) Affinity-purified rpn10-uim rpn13-KKD rpn1-D517A proteasomes contain reduced levels of Ddi1, Dsk2 and Ub conjugates in comparison to proteasomes from an rpn10-uim rpn13-KKD strain. Densitometric quantification of this blot is shown on the right. The amounts of UBL proteins were normalized to Rpn11FLAG and wild type levels were set as 100%. [file 1741-7007-9-33-S2.PDF]

Figure S3

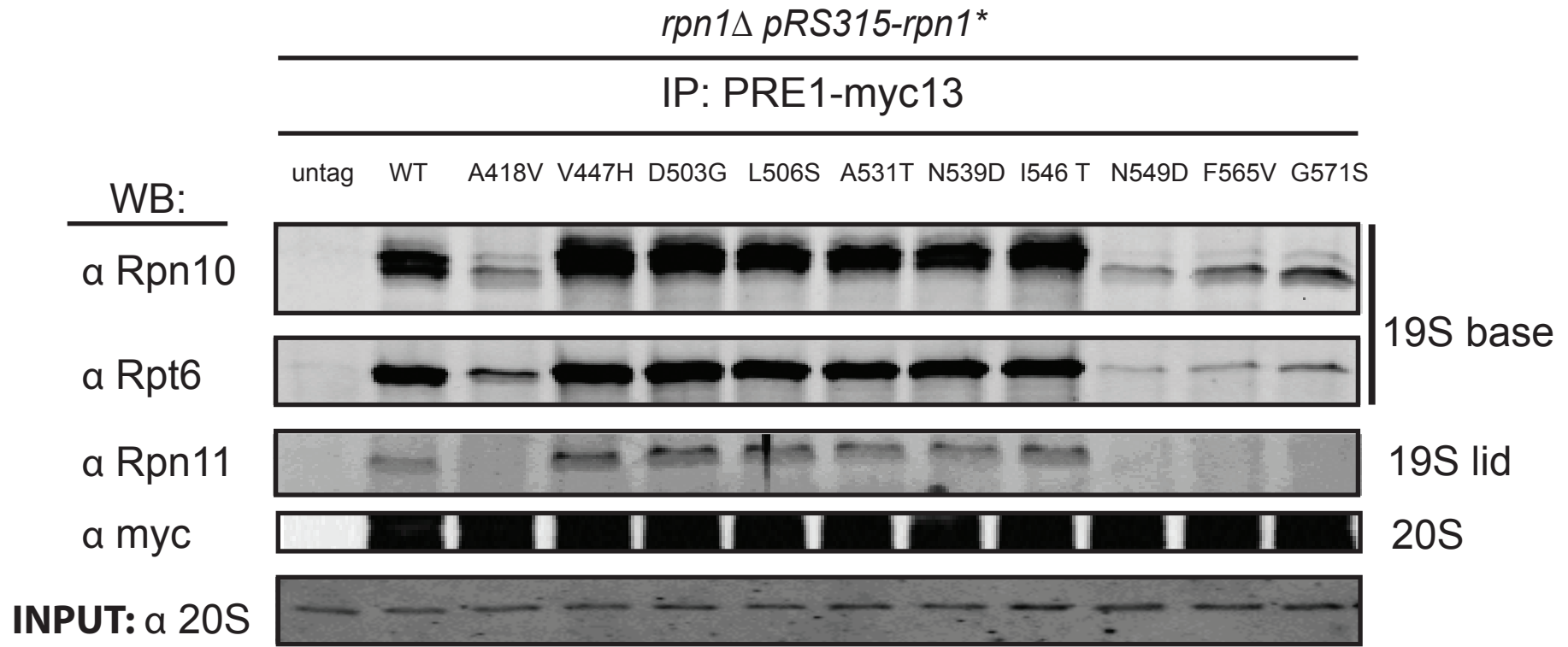

Supplement: Additional file 4 — Figure S3. Mutations at Rpn1 residues A418, N549, F565 and G571 render unstable proteasomes Pre1-myc13 tagged proteasomes from strains carrying plasmid borne Rpn1 alleles in an RPN1 null strain, were immunoprecipitated from whole cell extracts and analyzed by immunoblotting with the indicated antibodies. As shown, proteasomes with mutations at residues A418, N549, F565 and G571 exhibit dissociation of the 19S cap with the proteasomal base during immunoprecipitation experiments. [file 1741-7007-9-33-S4.PDF]

Figure S4

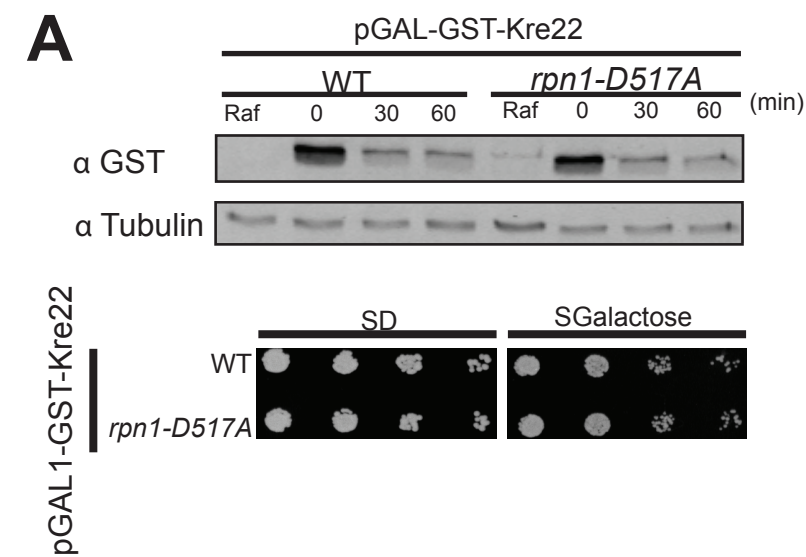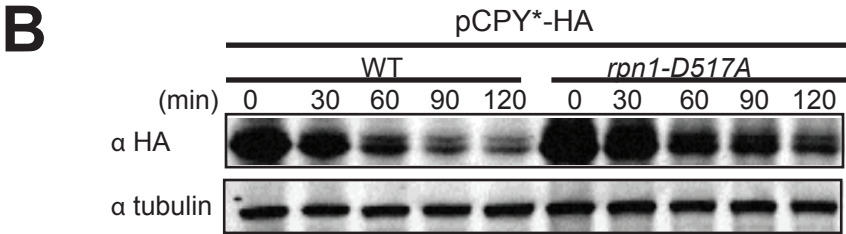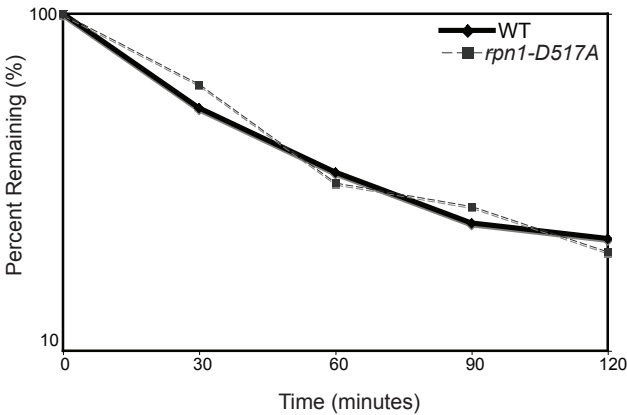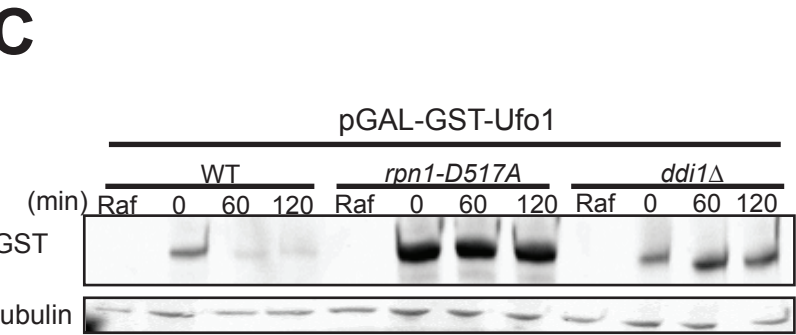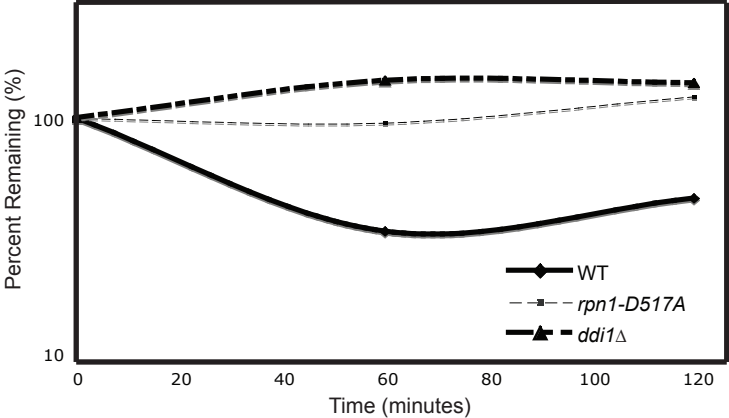

Supplement: Additional file 5 — Figure S4. rpn1-D517A mutants exhibit a selective defect in protein degradation. (A) Mutant rpn1-D517A cells degrade the Dsk2 substrate Kre22 with normal kinetics. Strains carrying a plasmid that expressed GST-Kre22 from the GAL1 promoter were grown in raffinose medium and then induced with 2% galactose for three hours. Dextrose was added at time zero to extinguish expression and samples were taken at the indicated time points for immunoblot analysis. Below, cells were plated in five-fold serial dilutions onto either glucosoe or galactose containing medium and monitored for growth after two to three days at 30°C. (B) Mutant rpn1-D517A cells degrade the Ufd1/Rad23/Dsk2 substrate CPY* with normal kinetics in a cycloheximide chase. Cycloheximide was added at time zero and samples were taken at the indicated time points for immunoblot analysis. Equal loading of extracts was confirmed by blotting with an anti-tubulin antibody (lower panel). The quantification of these blots is shown in the right panel. This is a replicate of the experiment shown in Figure 5A. (C) Ufo1 is stabilized in rpn1-D517A and ddi1Δ mutants. Wild type and mutant cells carrying a plasmid that expressed GST-Ufo1 from the GAL1 promoter were grown in raffinose medium and then induced with 2% galactose for 14 h. Dextrose was added at T0 to extinguish expression and samples were taken at the indicated time points and analyzed by immuoblot. Quantification is shown in the right-hand panel. This is a replicate of the experiment shown in Figure 5B [file 1741-7007-9-33-S5.PDF]
