# Supplementary material for: Identification of a functional docking site in the Rpn1 LRR domain for the UBA-UBL domain protein Ddi1
Source: BMC Biol. 2011 May 31;9:33. doi: 10.1186/1741-7007-9-33 (PMC3126750; doi:10.1186/1741-7007-9-33)
Supplement: Additional file 3 — Table S1. Additional rational Rpn1 mutants used in this study. Supplemental Table S2. S. cerevisiae strains used in this study. Supplemental Table S3. Plasmids used in this study. Supplemental Table S4. Antibodies used in this study [file 1741-7007-9-33-S3.PDF]

Additional Files

Supplemental Table 1. Additional Rational Rpn1 Mutants Used in this Study

Rpn1 Residue Mutation(s) [1]	Binds Ubp6	Binds Rad23	Binds Dsk2	Binds Ddi1
D482K	nd[2]	+	+	nd
T483R	nd	+	+	nd
D482K T483R K484A (KRA)	nd	+	+	nd
T516R	nd	+	+	nd
D517R	nd	+	+	nd
L518A	nd	+	+	nd
T516R D517R L518A (RRA)	nd			
I520E	nd	+	+	nd
E521K	nd	+	+	nd
T516R D517R L518A I520E E521K (RRAEK)	nd	+	+	nd
S500A T537A N539D (STN)	+[3]	+	+	+
D503G T537A N539D (DTN)	+	+	+	+
V447H D517A (VD)	+	+	+	-[4]
V447H K484A D517A (VKD)	+	+	+	-

[1]All listed Rpn1 mutant strains were tested for UBL binding competence in an *rpn13*Δ background
[2] "nd" indicates that no data was collected
[3] "+" indicates that binding was observed
[4] "-" indicates that no binding was observed

Supplemental Table 2. *S. cerevisiae* Strains Used in this Study

RJD #	Genotype	Source
4130	*MATa, leu2-3,112, trp1-901, his3D200, ade2-101, gal4D, gal80D, SPAL10::URA3, GAL1::lacZ, HIS3UAS GAL1::HIS3@LYS2, can1^R, cyh2^R (MaV203)*	Vidal Lab
4189	*Mata can1-100, leu2-3, his3-11,-15, trp1-1, ura3-1, ade2-1, rpn1::KanMX6 [pRS316-Rpn1-URA]*	this study
4626	*MATa leu2-3, his3-11,-15, trp1-1, ura3-1, ade2-1, rpn1::KanMX6 [pRS315-Rpn1-LEU]*	this study
4628	*MATa leu2-3, his3-11,-15, trp1-1, ura3-1, ade2-1, rpn1::KanMX6 [pRS315-Rpn1-V447H-LEU]*	this study
4748	*MATa leu2-3, his3-11,-15, trp1-1, ura3-1, ade2-1, rpn1::KanMX6 [pRS315-Rpn1-E466A-LEU]*	this study
4749	*MATa leu2-3, his3-11,-15, trp1-1, ura3-1, ade2-1, rpn1::KanMX6 [pRS315-Rpn1-N480A-LEU]*	this study
4750	*MATa leu2-3, his3-11,-15, trp1-1, ura3-1, ade2-1, rpn1::KanMX6 [pRS315-Rpn1-K484A-LEU]*	this study
4751	*MATa leu2-3, his3-11,-15, trp1-1, ura3-1, ade2-1, rpn1::KanMX6 [pRS315-Rpn1-S500A-LEU]*	this study
4629	*MATa leu2-3, his3-11,-15, trp1-1, ura3-1, ade2-1, rpn1::KanMX6 [pRS315-Rpn1-D503G-LEU]*	this study
4752	*MATa leu2-3, his3-11,-15, trp1-1, ura3-1, ade2-1, rpn1::KanMX6 [pRS315-Rpn1-D517A-LEU]*	this study
4633	*MATa leu2-3, his3-11,-15, trp1-1, ura3-1, ade2-1, rpn1::KanMX6 [pRS315-Rpn1-N539D-LEU]*	this study
4824	*MATa can1-100, leu2-3, his3-11,-15, trp1-1, ura3-1, ade2-1, rpn1::KanMX6 [pRS316-Rpn1-URA] rpn13::TRP1*	this study
5462	*MATa can1-100, leu2-3, his3-11,-15, trp1-1, ura3-1, ade2-1, rpn1::KanMX6 [pRS315-Rpn1-LEU] rpn13::TRP1*	this study
5463	*MATa can1-100, leu2-3, his3-11,-15, trp1-1, ura3-1, ade2-1, rpn1::KanMX6 [pRS315-Rpn1-K484A-LEU] rpn13::TRP1*	this study
5464	*MATa can1-100, leu2-3, his3-11,-15, trp1-1, ura3-1, ade2-1, rpn1::KanMX6 [pRS315-Rpn1-S500A-LEU] rpn13::TRP1*	this study
5465	*MATa can1-100, leu2-3, his3-11,-15, trp1-1, ura3-1, ade2-1, rpn1::KanMX6 [pRS315-Rpn1-D517A-LEU] rpn13::TRP1*	this study
5466	*MATa can1-100, leu2-3, his3-11,-15, trp1-1, ura3-1, ade2-1, rpn1::KanMX6 [pRS315-Rpn1-N539D-LEU] rpn13::TRP1*	this study
4920	*MATa leu2-3, his3-11,-15, trp1-1, ura3-1, ade2-1, rpn1::KanMX6 [pRS315-Rpn1-LEU] rpn13KKD::NatMX*	this study
4921	*MATa leu2-3, his3-11,-15, trp1-1, ura3-1, ade2-1, rpn1::KanMX6[pRS315-Rpn1-N480A-LEU] rpn13KKD::NatMX*	this study
4922	*MATa leu2-3, his3-11,-15, trp1-1, ura3-1, ade2-1, rpn1::KanMX6[pRS315-Rpn1-K484A-LEU] rpn13KKD::NatMX*	this study
4923	*MATa leu2-3, his3-11,-15, trp1-1, ura3-1, ade2-1, rpn1::KanMX6[pRS315-Rpn1-S500A-LEU] rpn13KKD::NatMX*	this study
4924	*MATa leu2-3, his3-11,-15, trp1-1, ura3-1, ade2-1, rpn1::KanMX6 [pRS315-Rpn1-D517A-LEU] rpn13KKD::NatMX*	this study
4925	*MATa leu2-3, his3-11,-15, trp1-1, ura3-1, ade2-1, rpn1::KanMX6[pRS315-Rpn1-N539D-LEU] rpn13KKD::NatMX*	this study
4926	*MATa leu2-3, his3-11,-15, trp1-1, ura3-1, ade2-1, rpn1::KanMX6 [pRS315-Rpn1-N480A-LEU] rpn10-UIM::KanMX*	this study
4927	*MATa leu2-3, his3-11,-15, trp1-1, ura3-1, ade2-1, rpn1::KanMX6[pRS315-Rpn1-K484A-LEU] rpn10-UIM::KanMX*	this study
4928	*MATa leu2-3, his3-11,-15, trp1-1, ura3-1, ade2-1, rpn1::KanMX6 [pRS315-Rpn1-S500A-LEU] rpn10-UIM::KanMX*	this study
4929	*MATa leu2-3, his3-11,-15, trp1-1, ura3-1, ade2-1, rpn1::KanMX6[pRS315-Rpn1-D517A-LEU] rpn10-UIM::KanMX*	this study
4930	*MATa leu2-3, his3-11,-15, trp1-1, ura3-1, ade2-1, rpn1::KanMX6 [pRS315-Rpn1-LEU] rpn13KKD::NatMX rpn10-UIM::KanMX*	this study
4931	*MATa leu2-3, his3-11,-15, trp1-1, ura3-1, ade2-1, rpn1::KanMX6 [pRS315-Rpn1-N480A-LEU] rpn13KKD::NatMX rpn10-UIM::KanMX*	this study

4932	*MATa leu2-3, his3-11,-15, trp1-1, ura3-1, ade2-1, rpn1::KanMX6 [pRS315-Rpn1-K484A-LEU] rpn13KKD::NatMX rpn10-UIM::KanMX*	this study
4933	*MATa leu2-3, his3-11,-15, trp1-1, ura3-1, ade2-1, rpn1::KanMX6[pRS315-Rpn1-S500A-LEU] rpn13KKD::NatMX rpn10-UIM::KanMX*	this study
4934	*MATa leu2-3, his3-11,-15, trp1-1, ura3-1, ade2-1, rpn1::KanMX6 [pRS315-Rpn1-D517A-LEU] rpn13KKD::NatMX rpn10-UIM::KanMX*	this study
4935	*MATa leu2-3, his3-11,-15, trp1-1, ura3-1, ade2-1, rpn1::KanMX6[pRS315-Rpn1-N539D-LEU] rpn13KKD::NatMX rpn10-UIM::KanMX*	this study
5106	*MATa leu2-3, his3-11,-15, trp1-1, ura3-1, ade2-1, rpn1::KanMX6 [pRS315-Rpn1-LEU] rpn4::TRP1*	this study
5107	*MATa leu2-3, his3-11,-15, trp1-1, ura3-1, ade2-1, rpn1::KanMX6[pRS315-Rpn1-N480A-LEU] rpn4::TRP1*	this study
5108	*MATa leu2-3, his3-11,-15, trp1-1, ura3-1, ade2-1, rpn1::KanMX6[pRS315-Rpn1-K484A-LEU] rpn4::TRP1*	this study
5109	*MATa leu2-3, his3-11,-15, trp1-1, ura3-1, ade2-1, rpn1::KanMX6[pRS315-Rpn1-S500A-LEU] rpnΔ::TRP1*	this study
5110	*MATa leu2-3, his3-11,-15, trp1-1, ura3-1, ade2-1, rpn1::KanMX6[pRS315-Rpn1-D517A-LEU] rpn4::TRP1*	this study
5111	*MATa leu2-3, his3-11,-15, trp1-1, ura3-1, ade2-1, rpn1::KanMX6 [pRS315-Rpn1-N539D-LEU] rpn4::TRP1*	this study
5459	*MATa leu2-3, his3-11,-15, trp1-1, ura3-1, ade2-1, rpn1::KanMX6 [pRS316-Rpn1-URA] rpn13::TRP1 arg4::KanMX lys2::HIS3 CAN1 rpn11::RPN11-FLAG-HphMX*	this study
5460	*MATa leu2-3, his3-11,-15, trp1-1, ura3-1, ade2-1, rpn1::KanMX6 [pRS315-Rpn1-K484A-LEU] rpn13::TRP1 arg4::KanMX lys2::HIS3 CAN1 rpn11::RPN11-FLAG-HphMX*	this study
5461	*MATa leu2-3, his3-11,-15, trp1-1, ura3-1, ade2-1, rpn1::KanMX6 [pRS315-Rpn1-D517A-LEU] rpn13::TRP1 arg4::KanMX lys2::HIS3 CAN1 rpn11::RPN11-FLAG-HphMX*	this study
5558	*MATa leu2-3, his3-11,-15, trp1-1, ura3-1, ade2-1, rpn1::KanMX6 [pRS315-Rpn1-A418V-LEU] rpn13::TRP1 arg4::KanMX lys2::HIS3 CAN1 rpn11::RPN11-FLAG-HphMX*	this study
5559	*MATa leu2-3, his3-11,-15, trp1-1, ura3-1, ade2-1, rpn1::KanMX6 [pRS315-Rpn1-V447H-LEU] rpn13::TRP1 arg4::KanMX lys2::HIS3 CAN1 rpn11::RPN11-FLAG-HphMX*	this study
5560	*MATa leu2-3, his3-11,-15, trp1-1, ura3-1, ade2-1, rpn1::KanMX6 [pRS315-Rpn1-E466A-LEU] rpn13::TRP1 arg4::KanMX lys2::HIS3 CAN1 rpn11::RPN11-FLAG-HphMX*	this study
5561	*MATa leu2-3, his3-11,-15, trp1-1, ura3-1, ade2-1, rpn1::KanMX6 [pRS315-Rpn1-N480A-LEU] rpn13::TRP1 arg4::KanMX lys2::HIS3 CAN1 rpn11::RPN11-FLAG-HphMX*	this study
5562	*MATa leu2-3, his3-11,-15, trp1-1, ura3-1, ade2-1, rpn1::KanMX6 [pRS315-Rpn1-S500A-LEU] rpn13::TRP1 arg4::KanMX lys2::HIS3 CAN1 rpn11::RPN11-FLAG-HphMX*	this study
5563	*MATa leu2-3, his3-11,-15, trp1-1, ura3-1, ade2-1, rpn1::KanMX6 [pRS315-Rpn1-* V447H K484A D517A *-LEU] rpn13::TRP1 arg4::KanMX lys2::HIS3 CAN1 rpn11::RPN11-FLAG-HphMX*	this study
4797	*MATa his3Δ1, leu2Δ0, met15Δ0, ura3Δ0, ddi1::KANMX*	Open Biosystems
5289	*MATA leu2-3, his3-11,-15, trp1-1, ura3-1, ade2-1, -LEU] rpn10-UIM::KanMX pre1::PRE1-FLAG-6XHIS-URA3*	this study
5290	*MATa leu2-3, his3-11,-15, trp1-1, ura3-1, ade2-1, rpn1::KanMX6 [pRS315-Rpn1 D517A-LEU rpn10-UIM::KanMX pre1::PRE1-FLAG-6XHIS-URA3*	this study
5457	*MATa leu2-3, his3-11,-15, trp1-1, ura3-1, ade2-1, rpn1::KanMX6 [pRS315-Rpn1 -LEU] rpn13KKD::NatMX rpn10-UIM::KanMX pre1::PRE1-FLAG-6XHIS-URA3*	this study
5458	*MATa leu2-3, his3-11,-15, trp1-1, ura3-1, ade2-1, rpn1::KanMX6 [pRS315-Rpn1 D517A-LEU] rpn13KKD::NatMX rpn10-UIM::KanMX pre1::PRE1-FLAG-6XHIS-URA3*	this study

Supplemental Table 3. Plasmids used in this study

Plasmid	Description	Source
RDB834	pGEX 4T-3	RJD Lab
RDB1647	pGEX6P1-Rad23	H. Yokosawa
RDB1672	pGEX-KG-Dsk2	M. Funakoshi
RDB2448	pET42a-Ddi1	H. Fu
RDB2170	pEXP22(AD)-Rad23	this study
RDB2173	pEXP22(AD)-Dsk2	this study
RDB2488	pEXP22(AD)-Ddi1	this study
RDB2490	pEXP22(AD)-Ddi1Ddi1ΔUBL (aa 78-428)	this study
RDB2179	pEXP22(AD)-Ubp6	this study
RDB2662	pEXP22(AD)-Rpn2	this study
RDB2115	pEXP32(DB)-Rpn1$^{391-642}$	this study
RDB2090	pRS316-Rpn1	this study
RDB2089	pRS315-Rpn1	this study
RDB2273	pRS315-Rpn1-V447H	this study
RDB2296	pRS315-Rpn1-E466A	this study
RDB2297	pRS315-Rpn1-N480A	this study
RDB2298	pRS315-Rpn1-K484A	this study
RDB2299	pRS315-Rpn1-S500A	this study
RDB2262	pRS315-Rpn1-D503G	this study
RDB2300	pRS315-Rpn1-D517A	this study
RDB2264	pRS315-Rpn1-N539D	this study
RDB2349	pRS315-Rpn1-S500A T537A N539D	this study
RDB2353	pRS315-Rpn1-D503G T537A N539D	this study
RDB2350	pRS315-Rpn1-V447H D517A	this study
RDB2354	pRS315-Rpn1-V447H K484A D517A	this study
RDB2407	pEGH-GAL-GST-Ufo1	OpenBiosystems
RDB2409	pEGH	Brenda Andrews
RDB1752	pCPY*HA/*URA3*	R. Hampton
RDB2408	pEGH-GAL-GST-Kre22	OpenBiosystems

Supplemental Table 4. Antibodies used in this study

Antibody	Source	
Rad23	Kiran Madura, Robert Wood Johnson Medical School	rabbit
Rad23	Santa Cruz (yG-20; sc-15556)	goat
Dsk2	Michael Glickman, Technion, Haifa, Israel	chicken
Dsk2	Abcam No.ab4119	rabbit
Ddi1	Jeffrey Gerst, Weizmann Institute	rabbit
Rpt5	Biomol No. PW8245	rabbit
Rpn3	Keiji Tanaka, Tokyo Metropolitan Inst. of Med. Sci.	rabbit
Rpn12	Daniel Finley, Harvard	rabbit
myc	Covance	mouse
flag	Sigma	mouse
Ub	Chemicon	mouse
Ub	Enzo	rabbit
Ubp6	Rohan Baker	rabbit

Supplemental Figure Legends

Figure S1. **Mutant *rpn1* alleles derived from both the RY2H screen and rational mutations display genetic interactions with mutations in genes that encode ubiquitin receptors intrinsic to the proteasome.**

Five-fold serial dilutions of cells were plated onto the indicated media. The *rpn1* mutants (*rpn1**) were plasmid shuffled into an *rpn1Δ* strain containing either no additional mutations (A) or *rpn13Δ (B)*. AZC refers to 5 mM of the proline analog l-azetidine-2-carboxylic acid (AZC). In panel B, mutations derived from the RY2H screen are indicated with a red box.

Figure S2. ***rpn1-D517A* , *rpn10-uim rpn1-D571A, and rpn10-uim rpn13-KKD rpn1-D571A* limit binding of UBA-UBL proteins Ddi1 and Dsk2**

(A) Affinity-purified *rpn1-D517A* proteasomes contain reduced levels of Ddi1 and Ub conjugates. Levels of UBA–UBL proteins, the lid subunit Rpn12 and polyubiquitin are shown for affinity purified proteasomes (IP) and in the whole cell extract input (WCE). (B) Affinity-purified *rpn10-uim rpn1-D517A* proteasomes similarly show diminished Ddi1 and Ub conjugates. (C) Affinity-purified *rpn10-UIM rpn13-KKD rpn1-D517A* proteasomes contain reduced levels of Ddi1, Dsk2 and Ub conjugates in comparison to proteasomes from a *rpn10-UIM rpn13-KKD* strain. Densitometric quantification of this blot is shown on the right. The amounts of UBL proteins were normalized to $Rpn11^{FLAG}$ and wild type levels were set as 100%.

Figure S3. **Mutations at Rpn1 residues A418, N549, F565 and G571 render unstable proteasomes**

Pre1-myc13 tagged proteasomes from strains carrying plasmid borne Rpn1 alleles in an *RPN1* null strain, were immunoprecipitated from whole cell extracts and analyzed by immunoblotting with the indicated antibodies. As shown, proteasomes with mutations at residues A418, N549, F565 and G571 exhibit dissociation of the 19S cap with the proteasomal base during immunoprecipitation experiments.

Figure S4. ***rpn1-D517A* mutants exhibit a selective defect in protein degradation.**

(A) Mutant *rpn1-D517A* cells degrade the Dsk2 substrate galactose inducible GST-Kre22 with normal kinetics. Strains carrying a plasmid that expressed GST-Kre22 from the GAL1 promoter were grown in raffinose medium and then induced with 2% galactose for 3 h. Dextrose was added at time zero to extinguish expression and samples were taken at the indicated time points. Below, cells were plated in a five-fold serial dilution onto either glucosoe or galactose containing media and monitored for growth after 2-3 days at 30°C. (B) Replicate of experiment seen in Figure 5A. Mutant *rpn1-D517A* cells degrade the Ufd1/Rad23/Dsk2 substrate CPY* with normal kinetics in a cycloheximide chase. Equal loading of extracts was confirmed by blotting with an anti-tubulin antibody (lower panel). The quantification of these blots is shown. (C) Replicate of experiment seen in Figure 5B. Ufo1 is stabilized in *rpn1-D517A* and *ddi1Δ* mutants. Wild type and mutant cells carrying a plasmid that expressed GST-Ufo1 from the GAL1 promoter were grown in raffinose medium and then induced with 2% galactose for 14 h. Dextrose was added at T_0 to extinguish expression and samples were taken at the indicated time points. Quantification is shown.
